# The putative mechanistic insights on how SARS-CoV-2 might influence the outcomes in cancer patients

**DOI:** 10.1186/s40164-022-00306-w

**Published:** 2022-09-07

**Authors:** Jingwen Deng, Xiaopeng Cai, Zhi Chen

**Affiliations:** 1 State Key Laboratory for Diagnosis and Treatment of Infectious Diseases, National Clinical Research Center for Infectious Diseases, National Medical Center for Infectious Diseases, Collaborative Innovation Center for Diagnosis and Treatment of Infectious Diseases, The First Affiliated Hospital, Zhejiang University School of Medicine, Hangzhou, 310003 China; 2 Department of Pathology, Key Laboratory of Disease Proteomics of Zhejiang Province, Zhejiang University School of Medicine, Hangzhou, 310058 China; 3 Department of Hepatobiliary and Pancreatic Surgery, The Second Affiliated Hospital Zhejiang University School of Medicine, Hangzhou, China

**Keywords:** SARS-CoV-2, COVID-19, Cancer, Prognosis, Genes, Proteins, Mechanism, Treatment

## Abstract

Early evidence indicated that cancer patients are at increased risk of adverse outcomes and mortality after severe acute respiratory syndrome coronavirus 2 (SARS-CoV-2) infection. To determine the putative mechanism by which SARS-CoV-2 affects patients with cancers, we conducted a preliminary exploration at the molecular level. We collected differentially expressed proteins (DEPs) in the lung, liver, kidney, and thyroid of postmortem coronavirus disease 2019 (COVID-19) and non-COVID-19 patients from iProX database. Furthermore, we collected differentially expressed genes (DEGs) related to overall survival (OS) in lung cancer, liver cancer, kidney cancer and thyroid cancer based on the Cancer Genome Atlas (TCGA) database. We obtained the intersection of DEPs and DEGs and identified the roles of shared and remaining DEPs in corresponding cancers based on published research. Finally, we found 192, 179, 154 and 147 DEPs in lung, liver, kidney and thyroid tissues and 486, 1140, 2245 and 31 DEGs related to OS in lung cancer, liver cancer, kidney cancer and thyroid cancer, respectively. 4, 8, 6 and 0 shared genes/proteins and 48, 42, 14 and 10 remaining proteins were verified to play a role in lung cancer, liver cancer, kidney cancer and thyroid cancer, respectively. Changes in 85% (44/52), 78% (39/50), 80% (16/20) and 90% (9/10) of the verified genes/proteins, including shared and remaining genes, showed poor effects on patients with the 4 cancer types with COVID-19. In conclusion, the changes in genes/proteins caused by SARS-CoV-2 might dictate the different degrees of adverse outcomes in patients with different tumors.

**Supplementary Information:**

The online version contains supplementary material available at 10.1186/s40164-022-00306-w.

To the editor,

Coronavirus disease 2019 (COVID-19) has spread on a global scale since it emerged in 2020, threatening the global economy and health. It is caused by severe acute respiratory syndrome coronavirus 2 (SARS-CoV-2), which mainly attacks the lungs and damages other organs [1]. Cancer patients have a high chance of exposure to SARS-CoV-2 due to regular testing and treatment in hospitals. Tumor-induced cachexia and treatment-induced immunosuppression make cancer patients generally vulnerable to SARS-CoV-2 [2]. To make matters worse, studies have found that cancer patients have more severe complications and higher mortality than the general population. It is thought-provoking that studies found no significantly increased risk of death in COVID-19 cancer patients receiving cancer treatment, and that patients with different cancers after SARS-CoV-2 infection have different prognoses. As a special group under the COVID-19 pandemic, cancer patients will experience these dramatic impacts until the crisis subsides. Therefore, personalized, evidenced cancer care and cancer treatment are critical for ensuring that cancer patients are treated safely without compromising overall outcomes.

Through careful bioinformatics mining, we found differentially expressed proteins (DEPs) caused by SARS-CoV-2 in 4 tissues and identified differentially expressed genes (DEGs) related to overall survival (OS) in these 4 cancers (Working pippelin in Fig. 1A). Confirmed by published literature, we found that the changes of 85%, 78%, 80% and 90% of the verified genes/proteins induced by SARS-CoV-2 had negative effects in lung cancer, liver cancer, kidney cancer and thyroid cancer patients with COVID-19 (Table 1, see Additional file 1 for detailed information). Because of the critical roles these genes/proteins play in tumors, we assumed that alterations in these genes/proteins are responsible for the poor prognosis of cancer patients infected with SARS-CoV-2 (Fig. 1B).

**Fig. 1:**
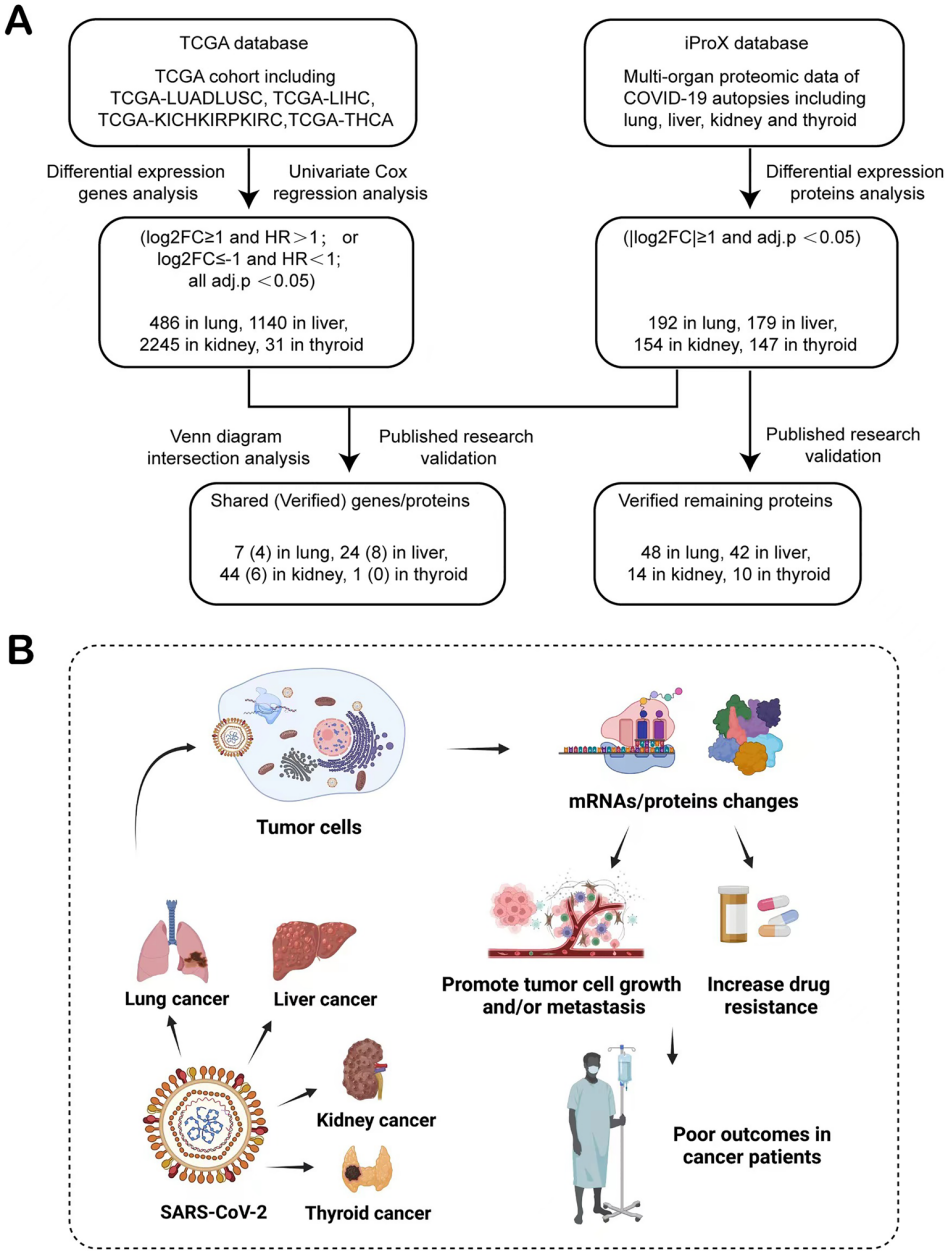
**A** Working pipeline. **B** Cancer patients with SARS-CoV-2 infection have adverse outcomes because of genes/proteins changes

**Table 1:** Verified genes/proteins based on the TCGA/HPA and iProX databases in lung, liver, kidney and thyroid

Tissues	Shared genes/proteins		Reminding genes/proteins	
	Changes showing poor effects	Changes showing non-poor effects	Changes showing poor effects	Changes showing non-poor effects
Lung	KRT6A, STEAP1, SLC7A5	SLC2A1	C1QBP, C5AR1, CALU, CKS2, CNPY2, COX5A, CTSB, CTSL, FKBP10, GFPT2, IRAK2, KRT17, METTL7B, MMP14, MRPL42, MTHFD2, NNMT, OAT, PFN2, PLIN2, PTX3, RCN1, S100P, SERPINE1, TNC, TREM1, YBX1, CTSA, FOSL2, IMP4, KRT14, LOXL1, PRDX3, PRDX4, SAA1, SCD, SFN, SOD2, CAVIN1, TMEM100, SELENBP1	PRDX2, SERPINB9, ALPL, FGA, GABARAPL1, RNF13, GLUL
Liver	S100P, SRXN1, RGN, PBLD, NDRG2, XDH, PDK4, TOP2A		BCL3, CD151, CD9, CHI3L1, FKBP5, FLOT2, GOLM1, GPX2, HK2, HRNR, IMPDH2, PFKFB3, PTGS2, RBM3, SERPINA3, SERPINE1, SPINK1, TSPAN8, TUFT1, BNIP3L, SDC4, THBS1, TSPAN31, ACY1, CA2, DPYSL3, GSTA1, PGM1, APOM, SEC14L2, CLEC4M	AHR, KAT7, MRC2, NUPR1, PGLS, POSTN, GALK1, GALNT2, NOLC1, SLFN11, B4GALT1
Kidney	NNMT, TGM2, ACY1, HAO2, PCK2, FBP1		ADAMTS1, CD151, HIF1A, JUNB, SAA1, SPP1, SOX9, NR4A1, NAMPT, DNPH1	SLC39A1, SPARCL1, TIMP3, ARG2
Thyroid			ALOX5, AXL, CXCL12, FKBP5, SLC34A2, STC1, FBN1, NRP2, HBB	APOA1

As one of the most common thoracic cancers, lung cancer appeared to worsen after the emergence of SARS-CoV-2 [3]. At the same time, recent studies also found that lung cancer patients have an increased probability of adverse outcomes after infection with SARS-CoV-2 compared with patients with other cancer types (55% vs. 28%) [4]. Consistent with these clinical features, we found more genes/proteins changes in the lung tissue of COVID-19 patients than in the liver, kidney and thyroid. Meanwhile, the number and ratio of genes/proteins that were verified to play a negative role in the progression of lung cancer were the highest among the 4 tumors we studied. We found a total of 192 DEPs in COVID-19 patients, and the changes in 44 (44/52, 85%) verified proteins played a negative role in lung cancer. These proteins not only mediate the malignant behavior of tumor cells but also affect chemotherapeutic drug sensitivity and the tumor immune microenvironment (see Additional file 1 for detailed information).

Because liver damage caused by COVID-19 complicates existing hepatitis virus infection and cirrhosis, HCC patients are more susceptible to the effects of SARS-CoV-2 than patients with other cancers [5]. A retrospective case–control analysis found that both patients with underlying liver cancer and patients recently diagnosed liver cancer had higher rates of SARS-CoV-2 infection than the general population (adjusted odds ratios were 12.5 and 6.5, respectively) [6]. In our work, we found that the putative underlying mechanism may be the changes in several genes/proteins. A total of 179 DEPs were found in COVID-19 patients, and changes in 39 (39/50, 78%) verified proteins had negative effects on liver cancer patients. In addition to affecting tumor cell proliferation, migration and invasion, these proteins affect the tumor immune microenvironment and tumor angiogenesis (see Additional file 1 for detailed information).

SARS-CoV-2 enters cells through the membrane protein ACE2, which is highly expressed in the kidney. Therefore, the kidney is also an important organ affected by SARS-CoV-2. However, there are few studies on the impact of the COVID-19 pandemic on kidney cancer. Wang et al. found that patients with kidney cancer had an adjusted odds ratio of 7.5 compared to the general population, which suggested an elevated infection rate [6]. Another small cohort study (17 patients) of the Russian Federation found that one-half of kidney cancer patients required hospitalization after SARS-CoV-2 infection, and 2 patients eventually died (2/17, 11%). They also found that interruption of anticancer treatment resulted in worsening performance status in half of the patients, which indicated that tumor progression in kidney cancer patients occurred during SARS-CoV-2 infection [7]. Our work showed that there were 154 DEPs in the kidney in COVID-19 patients, and 19 verified proteins were involved in kidney cancer progression. The changes in 16 (16/20, 80%) proteins showed negative effects on renal cell carcinoma and participated in the proliferation, migration and invasion of tumor cells (see Additional file 1 for detailed information).

SARS-CoV-2 was found to attack the pituitary-thyroid axis, which lead to new or recurrent thyroid dysfunction. A nationwide study in the United States found that the adjusted odds ratio of thyroid cancer patients infected with SARS-CoV-2 was 3.9, which was the lowest in 13 studied cancers [6]. Similarly, other studies found that thyroid cancer was not associated with the severity and poor prognosis of COVID-19, which indicated that thyroid cancer did not increase the mortality and morbidity of COVID-19 [8, 9]. Here, we found 147 DEPs in the thyroid of patients with SARS-CoV-2 infection, and changes of 9 (9/10, 90%) verified proteins were identified to play a negative role in thyroid cancer. These proteins are involved in thyroid cancer progression by affecting tumor cell proliferation and metastasis (see Additional file 1 for detailed information).

Overall, we found that SARS-CoV-2 might differentially affect the level of some genes/proteins, which are involved in tumor processes, in lung cancer, liver cancer, kidney cancer, and thyroid cancer. These genes/proteins enhance tumor proliferation, invasion and drug resistance behavior by altering the malignant and immune microenvironment of tumor. Collectively, our findings indicate that due to the changed pathophysiology of cancer tissues caused by SARS-CoV-2, cancer patients might experience tumor progression during or after SARS-CoV-2 infection. Fortunately, recent studies have found that immunotherapy [2, 10–12], targeted cancer therapy [2, 10–12], radiotherapy [10–12], hormone therapy [10–12] and chemotherapy [10–12] did not increase the risk of mortality in COVID-19 patients. Therefore, we suggest that the treatment of tumors should not be ignored during the treatment of COVID-19.

There are some exactly existing limitations of our study. First, few autopsy histopathological samples from COVID-19 patients were obtained, and these were from a Chinese cohort. Larger proteomic data from different countries, ethnicities, etc. are needed to jointly validate our findings. Second, whether unverified DEPs play a role in corresponding tumors needs further research. Of course, those non-DEPs that were filtered out cannot be ignored, perhaps because they are also involved in the tumor process. Finally, the patients enrolled predominantly had early variants of SARS-CoV-2 infection during early outbreaks. As virus strains mutate, these changes may also have different effects on tumors.

Most importantly, we should acknowledge the limitations of our analysis. We should fully consider the pre-existing medical conditions of the analyzed population when using TCGA database and iProX dataset. This concerns should include whether combined with other tumors, the cure status of tumors and the source of the control sample. First, as described in the article by Nie et al., P8 has gastric cancer, P30 has breast cancer, which should be excluded [13]. Actually, we used their tissue data from iProX dataset. On the one hand, it is because the iProX database has fewer samples, and on the other hand, it may be because the consideration of tumors in other parts has little effect on the target tumor. Second, we should consider the cure status of cancer patients with COVID-9. As described in the article by Nie et al., P4 and P12 had lung cancer, who provided lung tissue for biopsy [13]. However, we vaguely did not exclude the samples given the source of the autopsy sample (cancer or paracancer) and whether it has been cured. Third, we should get more information to exclude patients with tumors in the Non-COVID-9 group. Actually, Nie et al. recorded C26, C28 and C30 had kidney cancer, whose cure status and biopsy site were unclear [13]. Collectively, our data-mining study has some data analysis issues that require serious thought. But we consider it a research article that predicts how SARS-CoV-2 might affect cancer patients.

In summary, we have found some putative mechanistic insights on how SARS-CoV-2 might influence the outcomes in cancer patients. We suggest that antitumor treatment should be given to patients in a timely manner to prevent tumor recurrence and progression while fighting against COVID-19.

## Supplementary Information

**Additional file 1.** Detailed information for materials, methods and results.

## Data Availability

All data relevant to the study are included in the article or uploaded as supplementary information.

## Abbreviations

SARS-CoV-2: Severe acute respiratory syndrome coronavirus 2
COVID-19: Coronavirus disease 2019
DEPs: Differentially expressed proteins
DEGs: Differentially expressed genes
TCGA: The Cancer Genome Atlas
OS: Overall survival
