# Supplementary material for: The putative mechanistic insights on how SARS-CoV-2 might influence the outcomes in cancer patients
Source: Exp Hematol Oncol. 2022 Sep 7;11:52. doi: 10.1186/s40164-022-00306-w (PMC9449280; doi:10.1186/s40164-022-00306-w)
Supplement: Supplementary file 1 — Additional file 1. Detailed information for materials, methods and results. [file 40164_2022_306_MOESM1_ESM.docx]

**Additional information**

**The putative mechanistic insights on how SARS-CoV-2 might**

**influence the outcomes in cancer patients**

Jingwen Deng ^1,2^, Xiaopeng Cai ^3, *^, Zhi Chen^1, *^

^1^ State Key Laboratory for Diagnosis and Treatment of Infectious Diseases, National Clinical Research Center for Infectious Diseases, National Medical Center for Infectious Diseases, Collaborative Innovation Center for Diagnosis and Treatment of Infectious Diseases, The First Affiliated Hospital, Zhejiang University School of Medicine, Hangzhou 310003, China

^2^ Department of Pathology, Key Laboratory of Disease Proteomics of Zhejiang Province, Zhejiang University School of Medicine, Hangzhou 310058, China

^3^ Department of Hepatobiliary and Pancreatic Surgery, The Second Affiliated Hospital Zhejiang University School of Medicine, Hangzhou, China

*Correspondence authors.

E-mail: cxpeng@zju.edu.cn (Xiaopeng Cai); zjuchenzhi@zju.edu.cn (Zhi Chen).

**1. Materials and methods**

**1. Data collection**

We selected proteomics data of COVID-19 autopsies from the iProX database (Project ID: IPX0002393000) (<https://www.iprox.cn>). The original proteomic analysis of 134 autopsy samples from 4 organs in 19 COVID-19 patients and 56 non-COVID-19 patients ^[1]^. All control samples are histologically healthy tissue samples from the non-COVID-19 patients who were mostly victims of injuries. In case of lack of injured patients, benign lesions from cancer patients were procured. Clinical specimens are from the Union Hospital, Tongji Medical College, Huazhong University of Science and Technology. Here we selected the proteomics data of a total of 37 lung tissues (COVID-19: non-COVID-19=30:7), 36 liver tissues (COVID-19: non-COVID-19=28:8), 32 kidney tissues (COVID-19: non-COVID-19=21:11) and 29 thyroid tissues (COVID-19: non-COVID-19=14:15) proteomics for further analysis. Gene expression profiles and clinical information of patients were obtained from the Cancer Genome Atlas (TCGA) database (<https://portal.gdc.cancer.gov/>). RNA sequencing (RNA-seq) data of TCGA-LUADLUSC, TCGA-LIHC, TCGA-KICHKIRPKIRC and TCGA-THCA were extracted. Further, the samples with clinical data were screened for further analysis. Finally, a total of 1045 lung tissues (cancer: normal= 1037:108), 424 liver tissues (cancer:normal= 374:50), 1021 kidney tissues (cancer:normal= 893:128) and 568 thyroid tissues (cancer:normal= 510:58) were collected.

**2. Differential expression analysis**

We calculated the coefficient of variation by the log2 (abundance) of the quantified proteins in the control of 4 organs following the previous literature ^[1]^. Subsequently, the log 2 (fold change) (log2 FC) and adjusted p value (adj. p) were derived. Proteins with |log2 FC|≥1 and adj. p＜0.05 were considered DEPs. Next, DEG analysis based on RNA-seq data was performed using the DESeq2 R package^[2]^. Genes with |log2 FC|≥1 and adj. p＜0.05 were identified as DEGs.

**3. Gene‐related survival analysis and prediction biomarkers**

Univariate Cox regression analysis was employed to investigate the correlation between DEGs and patient OS. The survival and survminer packages were used for statistical analysis and the visualization of survival data, respectively ^[3]^. We used the median value method to define the high and low expression of genes: 0-50% was the low expression group, and >50% was the high expression group. Survival analysis corresponding to each gene was further performed. Typically, a difference between the low and high groups was considered statistically significant at p <0.05. We assumed that a hazard ratio (HR) >1 indicated potential biomarkers of poor prognosis, and HR<1 indicated potential biomarkers of good prognosis.

**4. Validation of protein expression data and published research**

First, we performed Venn diagram intersection analysis to select shared genes/proteins among the DEPs and OS-related DEGs. Next, we searched the PubMed, Embase and Web of Science databases to identify verified genes/proteins that were proven to have the exact function in corresponding cancers. Finally, the immunohistochemical data of the shared and verified genes/proteins were obtained from the Human Protein Atlas (HPA) database (<https://www.proteinatlas.org/>). However, we further searched for the remaining DEPs and clarified their role in corresponding cancers to avoid the omission of gene mining through the TCGA database. For example, the expression of some genes in liver cancer did not satisfy |log2FC|≥1, but these genes are involved in the development of cancer ^[4,5]^.

**2. Results:**

**1. DEPs and DEGs**

A total of 192, 179, 154 and 147 DEPs were found in the lung, liver, kidney and thyroid between COVID-19 and non-COVID-19 patients from the iProX database. Surprisingly, the number of upregulated proteins in the lungs were far greater than that of downregulated proteins (160>32), while the numbers of upregulated and downregulated proteins in the liver, kidney, and thyroid were roughly the same (**Additional table 1**). Based on the TCGA database, 6194, 4455, 11285 and 3187 DEGs were found in lung cancer, liver cancer, kidney cancer and thyroid cancer, respectively (**Additional table 2**). Among the 4 cancers, there were more upregulated DEGs than downregulated DEGs. These DEGs were further evaluated regarding their prognostic values.

**Additional table 1. Statistical summaries of COVID-19 autopsy dataset**

| Tissue source (COVID-19:non-COVID-19) | Raw proteins | Log2FC≥1 and adj.p＜0.05 | Log2FC≤-1 and adj.p＜0.05 |
| --- | --- | --- | --- |
| Lung (30:7) | 1606 | 160 | 32 |
| Liver (28:8) | 1969 | 95 | 84 |
| Kidney (21:11) | 1585 | 74 | 80 |
| Thyroid (14:15) | 1297 | 73 | 74 |

**Additional table 2. Statistical summaries of cancer comorbidities dataset**

| Dataset | Tissue source (cancer:normal) | Raw genes | Log2FC≥1 and  adj.p＜0.05 | Log2FC≤-1 and  adj.p＜0.05 | Log2FC≥1 and HR＞1 | Log2FC≤-1 and HR＜1 |
| --- | --- | --- | --- | --- | --- | --- |
| TCGA-LUADLUSC | Lung (1037:108) | 55060 | 3991 | 2203 | 286 | 200 |
| TCGA-LIHC | Liver (374:50) | 53674 | 3199 | 1256 | 895 | 245 |
| TCGA-KICHKIRPKIRC | Kidney (893:128) | 54992 | 6173 | 5112 | 1653 | 592 |
| TCGA-THCA | Thyroid (510:58) | 54483 | 1926 | 1261 | 22 | 9 |

LUAD, lung adenocarcinoma; LUSC, lung squamous cell carcinoma; LIHC, liver hepatocellular carcinoma; KICH, kidney chromophobe; KIRP, renal papillary cell carcinoma; KIRC, kidney renal clear cell carcinoma; THCA, thyroid carcinoma.

**2. Gene‐related survival analysis and prediction biomarkers**

We conducted univariate Cox regression analysis to identify prognostic DEGs and identified 486, 1140, 2245 and 31 genes related to OS in lung cancer, liver cancer, kidney cancer and thyroid cancer, respectively (**Additional table 2**). The gene HR was used to estimate the correlation between the gene expression level and OS. Finally, 286, 895, 1653 and 22 genes with high expression related to a shorter OS were obtained in the 4 cancers and were defined as potential biomarkers of poor prognosis; 200, 245, 592 and 9 genes with low expression related to a shorter OS were defined as potential biomarkers of good prognosis.

**3. Validation of protein expression data and published research**

We selected shared genes/proteins among the DEPs and OS-related DEGs. As shown in **Figure 1A**, we found 7, 24, 44 and 1 shared genes/proteins in lung, liver, kidney and thyroid tissues, respectively. Then, we identified 4, 8, 6 and 0 genes/proteins with exact functions in lung cancer, liver cancer, kidney cancer, and thyroid cancer in published research (**Table 1**). Finally, we evaluated the verified protein expression in tumor and normal tissues based on the HPA database.

Furthermore, we evaluated the role of the remaining DEPs in corresponding cancers and found 48, 42, 14 and 10 proteins with exact functions in lung cancer, liver cancer, kidney cancer and thyroid cancer (**Table 1**). The following sections describe the details of the verified genes/proteins in the 4 kinds of tumors.

**4. Genes/proteins in lung cancer**

As shown in **Additional table 3** and **AdditIonal figure 1**, 4 shared genes/proteins were verified and increased in lung cancer tissues. Highly expressed mRNA levels of these 4 genes were associated with poor OS in patients with lung cancer based on the TCGA and HPA databases. Meanwhile, research provided support that these 4 genes/proteins played a tumor-promoter role in lung cancer. They were highly expressed in lung cancer tissues and involved in the proliferation, migration and invasion of tumor cells, which led to a poor prognosis in lung cancer patients. Based on the iProX database, we found that 3 shared proteins (3/4, 75%) were more highly expressed after SARS-CoV-2 infection.

**Additional table 3. 4 shared and verified genes/proteins**

**based on the TCGA and iProX databases in lung**

| Genes/proteins-name | Role predictions based on the TCGA/HPA databases  (log2 FC) | Changes based on the iProX database (log2 FC) | Published research | | | |
| --- | --- | --- | --- | --- | --- | --- |
|  |  |  | mRNA/protein expression in lung cancer | Potential mechanism | Role in tumor | References |
| KRT6A | Biomarker of poor prognosis  （7.99） | 1.69 | Elevated | Promoted cell growth and migration via the EMT and MYC signaling | Tumor promoter | ^[6,7]^ |
| STEAP1 | Biomarker of poor prognosis  （3.03） | 1.63 | Elevated | Promoted cell proliferation, migration, and invasion via the EMT and JAK2/STAT3 pathways | Tumor promoter | ^[8,9]^ |
| SLC7A5 | Biomarker of poor prognosis  （3.17） | 1.00 | Elevated | Promoted cell proliferation | Tumor promoter | ^[10-12]^ |
| SLC2A1 | Biomarker of poor prognosis  （5.07） | -1.27 | Elevated | Promoted cell growth and migration | Tumor promoter | ^[13,14]^ |

**
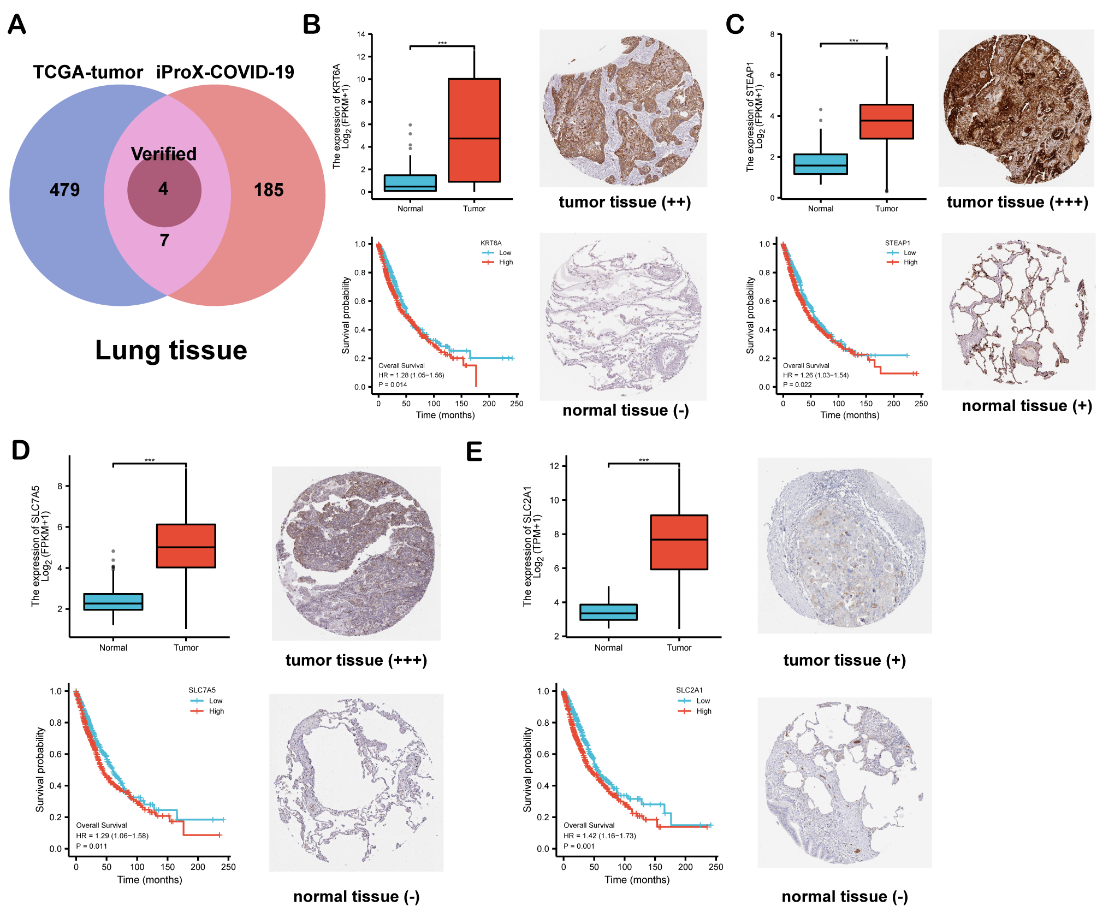
**

**Additional figure 1.** 4 shared and verified genes/proteins in the lung based on TCGA and iProX databasess. A. Venn diagram based on 486 OS-related DEGs and 192 DEPs in lung tissue. B-E. The mRNA/protein expression and survival curves of 4 verified genes/proteins based on the TCGA and HPA databases.

We identified 48 remaining DEPs that played a role in lung cancer based on published research. **Additional table 4** shows the details of the 48 remaining DEPs. 40 proteins were tumor promoters and participated in the occurrence of lung cancer by affecting tumor cell proliferation, migration or invasion. Among them, 13 proteins were upregulated in lung cancer tissues. 4 proteins, including CNPY2, CTSL, YBX1 and SFN, reduced the sensitivity to chemotherapy drugs. In addition, TNC inhibited tumor-infiltrating lymphocyte proliferation. We found that 38 (38/40, 95%) proteins had increased expression levels after SARS-CoV-2 infection. The other 8 proteins were tumor-suppressors. Among them, 3 proteins were downregulated in lung cancer tissues. These tumor suppressors inhibited tumor cell proliferation or metastasis. 3 proteins (3/8, 38%) were downregulated after SARS-CoV-2 infection.

Overall, the changes in 44 verified proteins (44/52, 85%) showed a negative effect on lung cancer patients with COVID-19.

**Additional table 4. 48 verified remaining proteins in the lung**

**based on the iProX database**

| Proteins-name | Changes based on the iProX database  (log2 FC) | Published research | | | |
| --- | --- | --- | --- | --- | --- |
|  |  | mRNA/protein expression in lung cancer | Potential mechanism | Role in tumor | References |
| C1QBP, C5AR1, CALU, CKS2, CNPY2, COX5A, CTSB, CTSL, FKBP10, GFPT2, IRAK2, KRT17, METTL7B, MMP14, MRPL42, MTHFD2, NNMT, OAT, PFN2, PLIN2, PTX3, RCN1, S100P, SERPINE1, TNC, TREM1, YBX1 | 1.37,  1.20,  1.07,1.43,  1.20,  1.08,  1.45,1.53,  1.55,  1.08,  1.33,  1.72,  1.12,  1.03,  1.10,  1.23,  1.39,1.03,  1.07,1.51,  1.43,1.29,  1.48,  1.48,  1.08,1.47,  1.22 | Elevated | Promoted tumor cell proliferation, migration or invasion;  CNPY2 increased resistance of cisplatin; CTSL mediated gefitinib, paclitaxel and cisplatin resistance; YBX1 reduced cisplatin sensitivity;  TNC inhibited tumor-infiltrating lymphocyte proliferation | Tumor promoter | ^[15,16]^ ^[17-19]^ ^[20]^ ^[21]^ ^[22-24]^ ^[25]^ ^[26]^ ^[27-29]^ ^[30]^ ^[31]^ ^[32]^ ^[33,34]^ ^[35,36]^ ^[37,38]^ ^[39]^ ^[40-42]^ ^[43-45]^ ^[46]^ ^[47,48]^ ^[49]^ ^[50-52]^ ^[53,54]^ ^[55]^ ^[56]^ ^[57,58]^ ^[59,60]^ ^[61,62]^ |
| CTSA, FOSL2, IMP4, KRT14, LOXL1, PRDX3, PRDX4, SAA1, SCD, SFN, SOD2 | 1.09，  1.49,1.25，  1.87，  1.13，  1.06，  1.15，  2.17,1.15，  2.17,1.26 | No mentioned | Promoted tumor cell proliferation, migration or invasion;  SFN increased cisplatin resistance | Tumor promoter | ^[63]^ ^[64,65]^ ^[66]^ ^[67,68]^ ^[69]^ ^[70]^ ^[71-73]^ ^[74]^ ^[75,76]^ ^[77-79]^ ^[80]^ |
| PRDX2, SERPINB9 | -1.81，  -1.06 | PRDX2 not mentioned; SERPINB9 elevated | Promoted tumor cell proliferation, migration or invasion | Tumor promoter | ^[81,82]^ ^[83]^ |
| CAVIN1, TMEM100, SELENBP1 | -1.20，  -1.45，  -1.09 | CAVIN1 and TMEM100 decreased; SELENBP1 not mentioned | Inhibited tumor cell proliferation, migration or invasion | Tumor suppressor | ^[84,85]^ ^[86-88]^ ^[89,90]^ |
| ALPL, FGA, GABARAPL1, RNF13, GLUL | 1.37，1.34，  1.01，  1.06，1.21 | ALPL and FGA decreased; the others not mentioned | Inhibited tumor cell migration or invasion;  GLUL increased the sensitivity of gefitinib and other drugs | Tumor suppressor | ^[91]^ ^[92]^  ^[93]^ ^[94]^ ^[95,96]^ |

**5. Genes/proteins in liver cancer**

8 shared genes/proteins were verified in liver tissue. 3 genes/proteins had increased expression levels and 5 genes/proteins had decreased expression levels in liver cancer tissue based on the TCGA and HPA databases (**Additional table 5** and **Additional figure 2**). The 3 upregulated proteins were associated with poor prognosis and identified as biomarkers of poor prognosis; the 5 downregulated proteins were associated with good prognosis and identified as biomarkers of good prognosis. Previous studies found that these 8 proteins affected the proliferation and migration of tumor cells in liver cancer. 3 proteins were upregulated and played a tumor-promoter role, and the other 5 proteins were downregulated and played a tumor-suppressor role in liver cancer. We found that all 8 shared proteins (8/8, 100%) had poor effects on liver cancer patients with COVID-19.

**Additional table 5. 8 shared and verified genes/proteins in the liver**

**based on TCGA and iProX databases**

| Genes/proteins-name | Role predictions based on the TCGA/HPA databases  (log2 FC) | Changes based on the iProX database  (log2 FC) | Published research | | | |
| --- | --- | --- | --- | --- | --- | --- |
|  |  |  | mRNA/protein expression in HCC | Potential mechanism | Role in tumor | References |
| S100P | Biomarker of poor prognosis  （5.69） | 1.84 | Elevated | Enhanced cell growth, migration, invasion and cytoskeleton arrangement | Tumor promoter | ^[97-99]^ |
| SRXN1 | Biomarker of poor prognosis  （1.38） | 1.04 | Elevated | Stimulated tumorigenesis and metastasis via the ROS/p65/BTG2 pathways | Tumor promoter | ^[100,101]^ |
| TOP2A | Biomarker of poor prognosis  （3.90） | 1.11 | Elevated | Enhanced cell growth, migration, invasion via EMT | Tumor promoter | ^[102,103]^ |
| RGN | Biomarker of poor prognosis  （-1.12） | -1.03 | Decreased | Affected prognosis through the apoptotic process, biological adhesion and blood coagulation; inhibited cell proliferation and invasion | Tumor suppressor | ^[104-106]^ |
| PBLD | Biomarker of good prognosis  （-1.78） | -1.15 | Decreased | Inhibited cell proliferation and invasion | Tumor suppressor | ^[107,108]^ |
| NDRG2 | Biomarker of good prognosis  （-1.52） | -1.51 | Decreased | Inhibited cancer cell adhesion, migration and invasion via CD24; prevented angiogenesis of HCC | Tumor suppressor | ^[109-111]^ |
| XDH | Biomarker of good prognosis  （-1.30） | -1.27 | Decreased | Inhibited cell migration and invasion via the EMT and TGFβ-Smad2/3 pathways | Tumor suppressor | ^[112]^ |
| PDK4 | Biomarker of good prognosis  （-1.23） | -1.08 | Decreased | Inhibited cell proliferation and migration by suppressing lipogenesis and mitochondrial energy metabolism | Tumor suppressor | ^[113-115]^ |


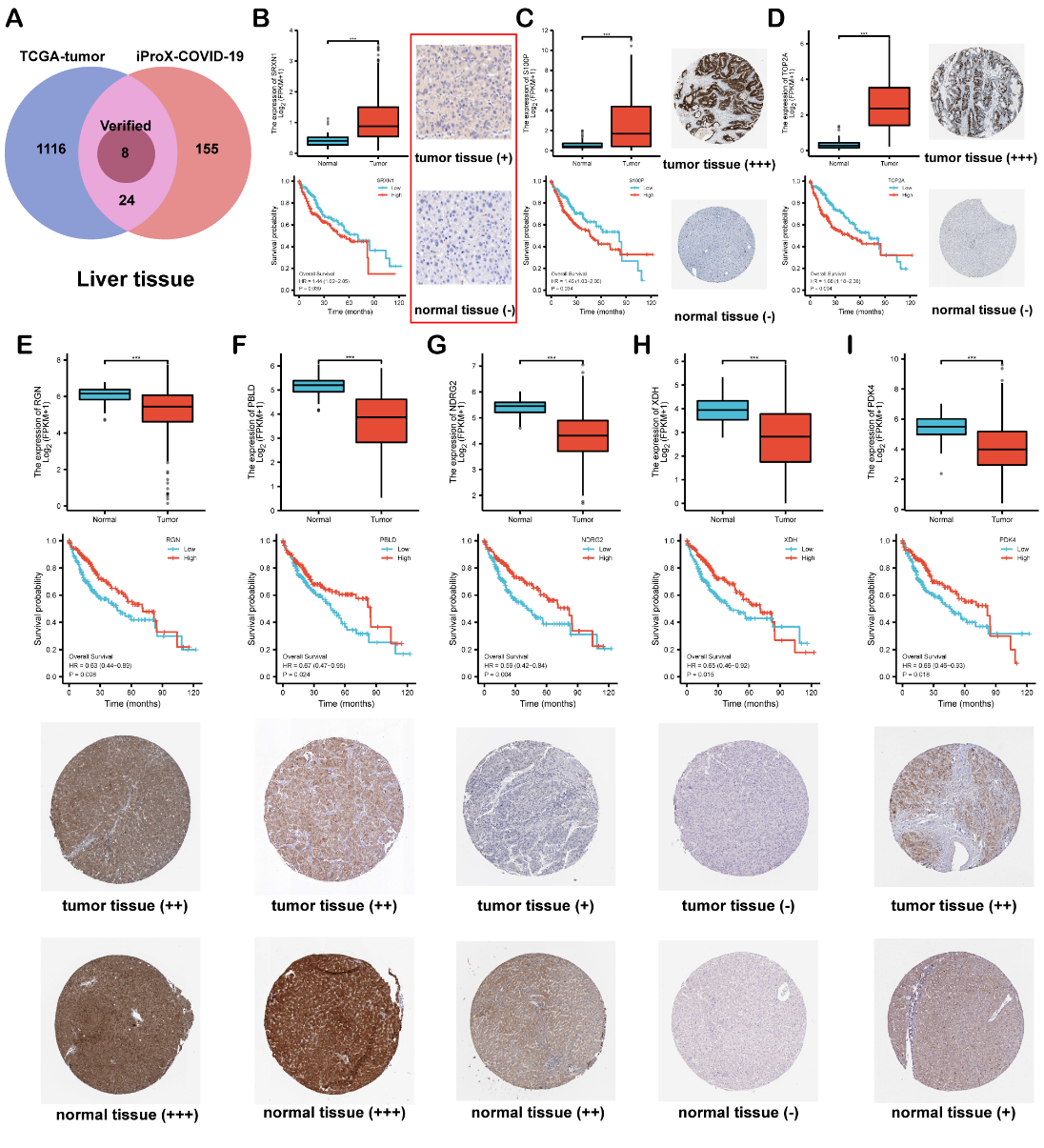


**Additional figure 2.** 8 shared and verified genes/proteins in the liver based on TCGA and iProX databases. A. Venn diagram based on 1140 OS-related DEGs and 179 DEPs in liver tissue. B-I. The mRNA/protein expression and survival curves of 8 verified genes/proteins based on the TCGA and HPA databases. The immunohistochemical staining data of SRXN1 was obtained from reference 101 ^[101]^.

As shown in **Additional table 6**, 42 remaining DEPs were verified to play a role in liver cancer. 30 proteins played a tumor-promoter role in liver cancer. In addition to affecting tumor cell proliferation, migration and invasion, 4 proteins (CD151, HK2, PFKFB3 and TSPAN8) also promoted the formation of tumor blood vessels. 2 proteins (FKBP5 and GOLM1) affected the tumor immune microenvironment by inhibiting the activity of NKT cells and CD8+ T cells. 23 proteins (23/30, 77%) were elevated after SARS-CoV-2 infection. There were 12 proteins that played a tumor-suppressor role in liver cancer. CLEC4M was identified as a potential tumor suppressor because it was positively associated with microvascular invasion and poor OS. These tumor suppressors inhibited the progression of liver cancer and showed decreased expression in liver cancer tissues. 8 proteins (8/12, 67%) showed decreased expression in COVID-19 patients.

Collectively, the changes in 39 verified proteins (39/50, 78%) showed negative effects on liver cancer patients with COVID-19.

**Additional table 6. 42 verified remaining proteins in the liver**

**based on iProX database**

| Proteins-name | Changes based on the iProX database  (log2 FC) | Published research | | | |
| --- | --- | --- | --- | --- | --- |
|  |  | mRNA/protein expression in HCC | Potential mechanism | Role in tumor | references |
| BCL3, CD151, CD9, CHI3L1, FKBP5, FLOT2, GOLM1, GPX2, HK2, HRNR, IMPDH2, PFKFB3, PTGS2, RBM3, SERPINA3, SERPINE1, SPINK1, TSPAN8, TUFT1 | 1.55，  1.14，  1.29，  1.95，  1.04，  1.21，  1.06，  1.42,1.50  1.24，  1.05，  1.12，  2.09，  1.37，  1.61，  1.97，  1.83，  1.13  1.22 | Elevated | Promoted tumor cell proliferation, migration or invasion;  CD151, HK2, PFKFB3 and TSPAN8 promoted neoangiogenesis;  FKBP5 and GOLM1 inhibited NKT cells and CD8+ T cells, respectively | Tumor promoter | ^[116,117]^ ^[118-121]^ ^[122]^ ^[123,124]^ ^[125]^ ^[126]^ ^[127-130]^ ^[131]^ ^[132,133]^ ^[134]^ ^[135]^ ^[136,137]^ ^[138-140]^ ^[141]^ ^[142]^ ^[143,144]^ ^[145,146]^ ^[147-149]^ ^[150,151]^ |
| BNIP3L, SDC4, THBS1, TSPAN31 | 1.02，  1.40，  1.25，  1.17 | No mentioned | Promoted tumor cell proliferation, migration or invasion;  BNIP3L increased cancer stemness | Tumor promoter | ^[152]^ ^[153]^ ^[154,155]^ ^[156]^ |
| AHR, KAT7, MRC2, NUPR1, PGLS, POSTN,  GALK1 | -1.03，  -1.08，  -1.58，  -1.01，  -1.14，  -1.41 | Elevated,  GALK1 not mentioned | Promoted tumor cell proliferation, migration or invasion | Tumor promoter | ^[157]^ ^[158]^ ^[159]^ ^[160]^ ^[161]^ ^[162,163]^ ^[164]^ |
| ACY1,  CA2, DPYSL3, GSTA1, PGM1 | -1.01，  -1.17，  -1.06，  -1.51，  -1.03 | Decreased | Inhibited tumor cell proliferation, migration or invasion | Tumor suppressor | ^[165]^ ^[166,167]^ ^[168]^ ^[169]^ ^[170]^ |
| APOM，  SEC14L2 | -1.20，  -1.05 | No mentioned | Inhibited tumor cell growth or metastasis | Tumor suppressor | ^[171]^ ^[172]^ |
| GALNT2, NOLC1, SLFN11,  B4GALT1 | 1.21，  1.05，  1.02，  1.10 | Decreased | Inhibited tumor cell proliferation, migration or invasion | Tumor suppressor | ^[173]^ ^[174]^ ^[175]^ ^[176]^ |
| CLEC4M | -2.20 | Decreased | Inhibited cell proliferation and enhanced apoptosis via the STAT3 pathway | Contradictory | ^[177,178]^ |

**6. Genes/proteins in kidney cancer**

As shown in **Additional table 7** and **Additional figure 3**, 6 shared genes/proteins were verified in the kidney. 2 genes/proteins were upregulated, and 4 genes/proteins were downregulated in kidney cancer tissue based on the TCGA and HPA databases. Based on published research, 2 upregulated proteins played a tumor-promoter role, and 4 downregulated proteins played a tumor-suppressor role in kidney cancer. Among them, PCK2 increased sensitivity to sunitinib and inhibited kidney cancer malignancy. The expression of 6 proteins (6/6, 100%) changed after SARS-CoV-2 infection, which promoted the progression of kidney cancer.

**Additional table 7. 6 shared and verified genes/proteins in the kidney**

**based on TCGA and iProX databases**

| Genes/proteins-name | Role predictions based on the TCGA/HPA databases  (log2 FC) | Changes based on the iProX database  (log2 FC) | Published research | | | |
| --- | --- | --- | --- | --- | --- | --- |
|  |  |  | mRNA/protein expression in kidney cancer | Potential mechanism | Role in tumor | References |
| NNMT | Biomarker of poor prognosis  （3.23） | 1.9408 | Elevated | Promoted cell invasion via the PI3K/Akt/SP1/MMP-2 pathways | Tumor promoter | ^[179-181]^ |
| TGM2 | Biomarker of poor prognosis  （1.29） | 1.4149 | Elevated in RCC cell lines | Upregulation involved in the development and metastasis of tumor cell | Tumor promoter | ^[182]^ |
| ACY1 | Biomarker of good prognosis  （-1.29） | -1.1093 | Decreased in rat RCC | Inhibited cell growth and colony formation | Tumor suppressor | ^[183]^ |
| HAO2 | Biomarker of good prognosis  （-8.56） | -1.0086 | Decreased | Inhibited malignancy by promoting the lipid catabolic process | Tumor suppressor | ^[184]^ |
| PCK2 | Biomarker of good prognosis  （-1.39） | -1.0898 | Decreased | Inhibited tumor cell malignancy and increased sensitivity to sunitinib by regulating ERS | Tumor suppressor | ^[185]^ |
| FBP1 | Biomarker of good prognosis  （-2.28） | -1.1715 | Elevated | Inhibited tumor cell by antagonizing glycolytic flux and HIF function | Tumor suppressor | ^[186,187]^ |


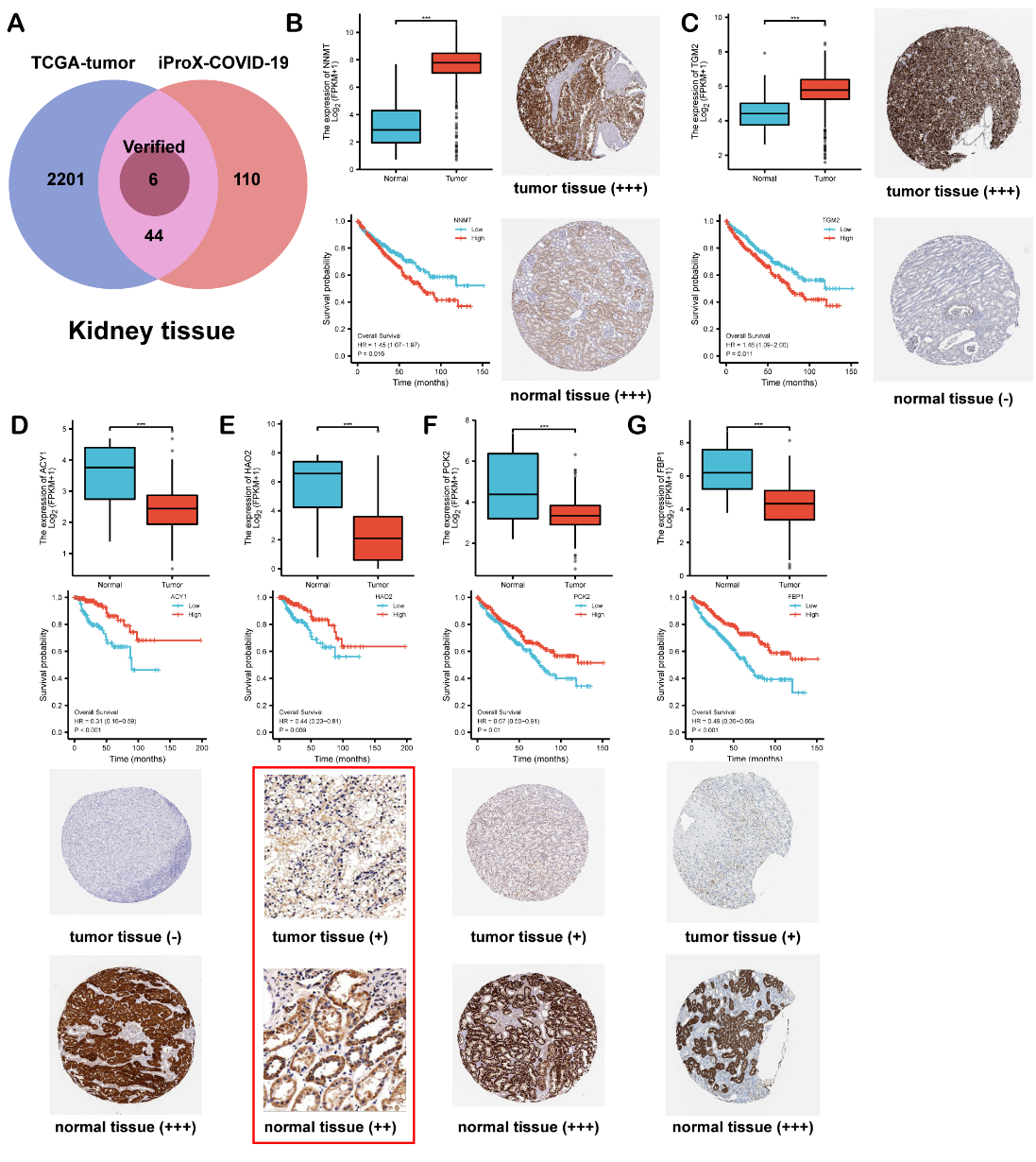


**Additional figure 3.** 6 shared and verified genes/proteins in the kidney based on TCGA and iProX databases. A. Venn diagram based on 2245 OS-related DEGs and 154 DEPs in kidney tissue. B-G. The mRNA/protein expression and survival curves of 6 verified genes/proteins based on the TCGA and HPA databases. The immunohistochemical staining data of HAO2 was obtain from reference 184 ^[184]^.

14 remaining DEPs were verified to take part in the progression of kidney cancer (**Addtional table 8**). These 14 proteins directly affected the occurrence of kidney cancer by participating in the proliferation, migration and invasion of tumor cells. Among them, 9 upregulated proteins played a tumor-promoter role, and 5 downregulated proteins played a tumor-suppressor role in kidney cancer. Only DNPH1, as a tumor suppressor, were decreased in COVID-19 patients.

Finally, we found that the changes of 16 proteins (16/20, 80%) showed a negative effect on kidney cancer patients with COVID-19.

**Additional table 8. 14 verified remaining proteins in the kidney**

**based on iProX database**

| Proteins-name | Changes based on the iProX database  (log2 FC) | Published research | | | |
| --- | --- | --- | --- | --- | --- |
|  |  | mRNA/protein expression in kidney cancer | Potential mechanism | Role in tumor | references |
| ADAMTS1，CD151，HIF1A，JUNB，SAA1，SPP1，SOX9 | 1.25，  1.13，  1.85，  1.26，  1.33，  1.30，  1.09 | Elevated | Promoted tumor cell proliferation, migration or invasion；  JUNB promoted angiogenesis;  SOX9 was involved in TKIs resistance via the Raf/MEK/ERK pathways | Tumor promoter | ^[188]^ ^[189,190]^ ^[191-193]^ ^[194]^ ^[195]^ ^[196-198]^ ^[199,200]^ |
| NR4A1, NAMPT | 1.35，  1.12 | No menntioned | Promoted tumor cell growth | Tumor promoter | ^[201]^ ^[202]^ |
| DNPH1 | -1.02 | Decreased | Inhibited tumor cell proliferation | Tumor suppressor | ^[203]^ |
| SLC39A1, SPARCL1 | 1.62，  1.08 | Decreased | Inhibited tumor cell proliferation, migration or invasion | Tumor suppressor | ^[204]^ ^[205]^ |
| TIMP3, ARG2 | 1.32，  1.21 | No mentioned | TIMP3 inhibited cell migration and invasion;  ARG2 suppressed RCC by depleting biosynthetic cofactor pyridoxal phosphate and Increasing polyamine toxicity | Tumor suppressor | ^[206-208]^ ^[209]^ |

**7. Genes/proteins in thyroid cancer**

We found that F12 was the only shared gene/protein in the thyroid based on the TCGA and HPA databases (**Additional table 9 and Additional figure 4**). We assumed that F12 was a poor biomarker because its high expression was associated with a poor prognosis in thyroid cancer. However, there is no published research on F12 to verify its function in thyroid cancer.

**Additional table 9. Shared F12 gene/protein in the thyroid**

**based on TCGA and iProX databases**

| Genes/proteins-name | Role predictions based on the TCGA/HPA databases  (log2 FC) | Changes based on the iProX database  (log2 FC) | Published research | | | |
| --- | --- | --- | --- | --- | --- | --- |
|  |  |  | mRNA/protein expression in thyroid cancer | Potential mechanism | Role in tumor | References |
| F12 | Biomarker of good prognosis  （1.38） | -1.72 | - | - | - | None |


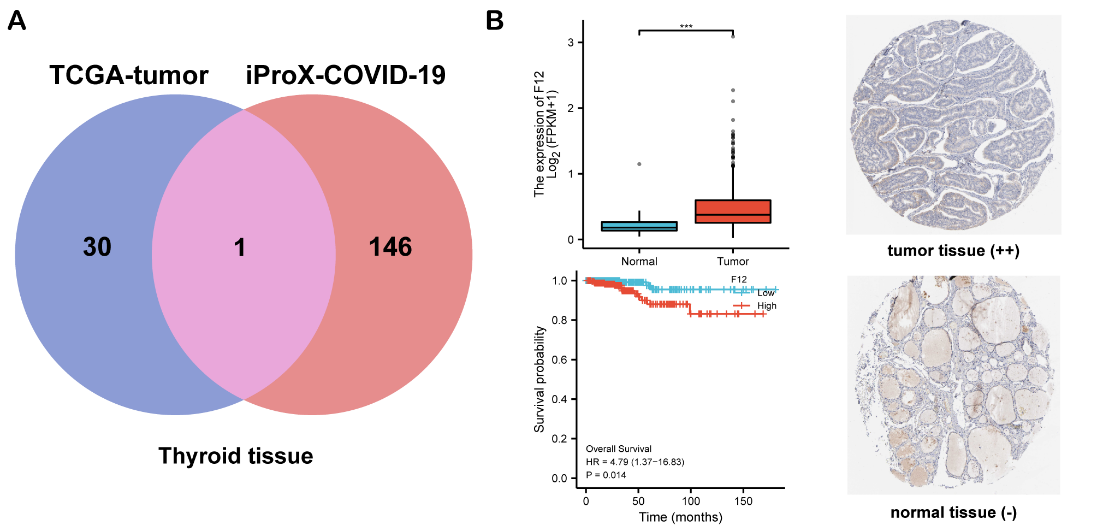


**Additional figure 4.** 1 shared gene/protein in the thyroid based on TCGA and iProX databases. A. Venn diagram based on 31 OS-related DEGs and 147 DEPs in thyroid tissue. B. The mRNA/protein expression and survival curves of F12 based on the TCGA and HPA databases.

As shown in **Additional table 10**, 10 remaining DEPs were verified to play promoter/suppressor roles in thyroid cancer. 9 proteins were upregulated in cancer tissues and were verified as tumor promoters. Except for APOA1, the other 8 proteins were all elevated after SARS-CoV-2 infection.1 protein (HBB) was downregulated and was verified as a tumor suppressor. Finally, we found that the changes in 9 proteins (9/10, 90%) showed a negative effect on thyroid cancer patients with COVID-19.

**Additional table 10. 10 verified remaining proteins in the thyroid**

**based on iProX database**

| Proteins-name | Changes based on the iProX database  (log2 FC) | Published research | | | |
| --- | --- | --- | --- | --- | --- |
|  |  | mRNA/protein expression in PTC | Potential mechanism | Role in tumor | References |
| ALOX5，AXL，CXCL12，FKBP5，SLC34A2，STC1 | 1.03，  1.51，  1.31，  1.12，  1.17，  1.68 | Elevated | Promoted tumor cell proliferation, migration or invasion | Tumor promoter | ^[210]^ ^[211-213]^ ^[214-216]^ ^[217]^ ^[218]^ ^[219,220]^ |
| FBN1, NRP2 | 1.02，  1.21 | No mentioned | FBN1 was involved in tumor cell proliferation and apoptosis;  NRP2 promoted tumor cell proliferation, migration, and invasion | Tumor promoter | ^[221]^ ^[222-224]^ |
| APOA1 | -2.65 | Elevated | Promoted tumor cell migration and invasion | Tumor promoter | ^[225]^ |
| HBB | -2.97 | Decreased in anaplastic thyroid cancer | Suppressed KTA2 growth | Tumor suppressor | ^[226]^ |

**References:**

[1] Nie X, Qian L, Sun R, et al. Multi-organ proteomic landscape of COVID-19 autopsies[J]. Cell, 2021, 184(3): 775-791.e714.

[2] Love MI, Huber W, Anders S. Moderated estimation of fold change and dispersion for RNA-seq data with DESeq2[J]. Genome Biol, 2014, 15(12): 550.

[3] Liu J, Lichtenberg T, Hoadley KA, et al. An Integrated TCGA Pan-Cancer Clinical Data Resource to Drive High-Quality Survival Outcome Analytics[J]. Cell, 2018, 173(2): 400-416.e411.

[4] Cai X, Deng J, Zhou J, et al. Cyclin-dependent kinase 19 upregulation correlates with an unfavorable prognosis in hepatocellular carcinoma[J]. BMC Gastroenterol, 2021, 21(1): 377.

[5] Cai X, Zhou J, Deng J, et al. Prognostic biomarker SMARCC1 and its association with immune infiltrates in hepatocellular carcinoma[J]. Cancer Cell Int, 2021, 21(1): 701.

[6] Yang B, Zhang W, Zhang M, et al. KRT6A Promotes EMT and Cancer Stem Cell Transformation in Lung Adenocarcinoma[J]. Technol Cancer Res Treat, 2020, 19: 1533033820921248.

[7] Che D, Wang M, Sun J, et al. KRT6A Promotes Lung Cancer Cell Growth and Invasion Through MYC-Regulated Pentose Phosphate Pathway[J]. Front Cell Dev Biol, 2021, 9: 694071.

[8] Huo SF, Shang WL, Yu M, et al. STEAP1 facilitates metastasis and epithelial-mesenchymal transition of lung adenocarcinoma via the JAK2/STAT3 signaling pathway[J]. Biosci Rep, 2020, 40(6).

[9] Guo Q, Ke XX, Liu Z, et al. Evaluation of the Prognostic Value of STEAP1 in Lung Adenocarcinoma and Insights Into Its Potential Molecular Pathways via Bioinformatic Analysis[J]. Front Genet, 2020, 11: 242.

[10] Miko E, Margitai Z, Czimmerer Z, et al. miR-126 inhibits proliferation of small cell lung cancer cells by targeting SLC7A5[J]. FEBS Lett, 2011, 585(8): 1191-1196.

[11] Wang J, Ding M, Zhu H, et al. Up-regulation of long noncoding RNA MINCR promotes non-small cell of lung cancer growth by negatively regulating miR-126/SLC7A5 axis[J]. Biochem Biophys Res Commun, 2019, 508(3): 780-784.

[12] Li H, Chen S, Liu J, et al. Long non-coding RNA PVT1-5 promotes cell proliferation by regulating miR-126/SLC7A5 axis in lung cancer[J]. Biochem Biophys Res Commun, 2018, 495(3): 2350-2355.

[13] Guo W, Sun S, Guo L, et al. Elevated SLC2A1 Expression Correlates with Poor Prognosis in Patients with Surgically Resected Lung Adenocarcinoma: A Study Based on Immunohistochemical Analysis and Bioinformatics[J]. DNA Cell Biol, 2020, 39(4): 631-644.

[14] Wang Y, Shi S, Ding Y, et al. Metabolic reprogramming induced by inhibition of SLC2A1 suppresses tumor progression in lung adenocarcinoma[J]. Int J Clin Exp Pathol, 2017, 10(11): 10759-10769.

[15] Li W, Zhang X, Wang W, et al. Quantitative proteomics analysis of mitochondrial proteins in lung adenocarcinomas and normal lung tissue using iTRAQ and tandem mass spectrometry[J]. Am J Transl Res, 2017, 9(9): 3918-3934.

[16] Kim KB, Yi JS, Nguyen N, et al. Cell-surface receptor for complement component C1q (gC1qR) is a key regulator for lamellipodia formation and cancer metastasis[J]. J Biol Chem, 2011, 286(26): 23093-23101.

[17] Ajona D, Zandueta C, Corrales L, et al. Blockade of the Complement C5a/C5aR1 Axis Impairs Lung Cancer Bone Metastasis by CXCL16-mediated Effects[J]. Am J Respir Crit Care Med, 2018, 197(9): 1164-1176.

[18] Ajona D, Ortiz-Espinosa S, Moreno H, et al. A Combined PD-1/C5a Blockade Synergistically Protects against Lung Cancer Growth and Metastasis[J]. Cancer Discov, 2017, 7(7): 694-703.

[19] Gu J, Ding JY, Lu CL, et al. Overexpression of CD88 predicts poor prognosis in non-small-cell lung cancer[J]. Lung Cancer, 2013, 81(2): 259-265.

[20] Nagano K, Imai S, Zhao X, et al. Identification and evaluation of metastasis-related proteins, oxysterol binding protein-like 5 and calumenin, in lung tumors[J]. Int J Oncol, 2015, 47(1): 195-203.

[21] Wan Z, Wang L, Yang D, et al. CKS2 Promotes the Growth in Non-Small-Cell Lung Cancer by Downregulating Cyclin-Dependent Kinase Inhibitor[J]. Pathobiology, 2021: 1-10.

[22] Wang H, Kanmangne D, Li R, et al. miR‑30a‑3p suppresses the proliferation and migration of lung adenocarcinoma cells by downregulating CNPY2[J]. Oncol Rep, 2020, 43(2): 646-654.

[23] Dou Y, Lei JQ, Guo SL, et al. The CNPY2 enhances epithelial-mesenchymal transition via activating the AKT/GSK3β pathway in non-small cell lung cancer[J]. Cell Biol Int, 2018, 42(8): 959-964.

[24] Yu D, Qin Y, Jun-Qiang L, et al. CNPY2 enhances resistance to apoptosis induced by cisplatin via activation of NF-κB pathway in human non-small cell lung cancer[J]. Biomed Pharmacother, 2018, 103: 1658-1663.

[25] Chen WL, Kuo KT, Chou TY, et al. The role of cytochrome c oxidase subunit Va in non-small cell lung carcinoma cells: association with migration, invasion and prediction of distant metastasis[J]. BMC Cancer, 2012, 12: 273.

[26] Gong F, Peng X, Luo C, et al. Cathepsin B as a potential prognostic and therapeutic marker for human lung squamous cell carcinoma[J]. Mol Cancer, 2013, 12(1): 125.

[27] Han ML, Zhao YF, Tan CH, et al. Cathepsin L upregulation-induced EMT phenotype is associated with the acquisition of cisplatin or paclitaxel resistance in A549 cells[J]. Acta Pharmacol Sin, 2016, 37(12): 1606-1622.

[28] Cui F, Wang W, Wu D, et al. Overexpression of Cathepsin L is associated with gefitinib resistance in non-small cell lung cancer[J]. Clin Transl Oncol, 2016, 18(7): 722-727.

[29] Zhao Y, Shen X, Zhu Y, et al. Cathepsin L-mediated resistance of paclitaxel and cisplatin is mediated by distinct regulatory mechanisms[J]. J Exp Clin Cancer Res, 2019, 38(1): 333.

[30] Ramadori G, Ioris RM, Villanyi Z, et al. FKBP10 Regulates Protein Translation to Sustain Lung Cancer Growth[J]. Cell Rep, 2020, 30(11): 3851-3863.e3856.

[31] Szymura SJ, Zaemes JP, Allison DF, et al. NF-κB upregulates glutamine-fructose-6-phosphate transaminase 2 to promote migration in non-small cell lung cancer[J]. Cell Commun Signal, 2019, 17(1): 24.

[32] Seol HS, Akiyama Y, Shimada S, et al. Epigenetic silencing of microRNA-373 to epithelial-mesenchymal transition in non-small cell lung cancer through IRAK2 and LAMP1 axes[J]. Cancer Lett, 2014, 353(2): 232-241.

[33] Wang Z, Yang MQ, Lei L, et al. Overexpression of KRT17 promotes proliferation and invasion of non-small cell lung cancer and indicates poor prognosis[J]. Cancer Manag Res, 2019, 11: 7485-7497.

[34] Liu J, Liu L, Cao L, et al. Keratin 17 Promotes Lung Adenocarcinoma Progression by Enhancing Cell Proliferation and Invasion[J]. Med Sci Monit, 2018, 24: 4782-4790.

[35] Ali J, Liu W, Duan W, et al. METTL7B (methyltransferase-like 7B) identification as a novel biomarker for lung adenocarcinoma[J]. Ann Transl Med, 2020, 8(18): 1130.

[36] Liu D, Li W, Zhong F, et al. METTL7B Is Required for Cancer Cell Proliferation and Tumorigenesis in Non-Small Cell Lung Cancer[J]. Front Pharmacol, 2020, 11: 178.

[37] Stawowczyk M, Wellenstein MD, Lee SB, et al. Matrix Metalloproteinase 14 promotes lung cancer by cleavage of Heparin-Binding EGF-like Growth Factor[J]. Neoplasia, 2017, 19(2): 55-64.

[38] Xu M, Wang YZ. miR‑133a suppresses cell proliferation, migration and invasion in human lung cancer by targeting MMP‑14[J]. Oncol Rep, 2013, 30(3): 1398-1404.

[39] Jiang W, Zhang C, Kang Y, et al. MRPL42 is activated by YY1 to promote lung adenocarcinoma progression[J]. J Cancer, 2021, 12(8): 2403-2411.

[40] Shi Y, Xu Y, Yao J, et al. MTHFD2 promotes tumorigenesis and metastasis in lung adenocarcinoma by regulating AKT/GSK-3β/β-catenin signalling[J]. J Cell Mol Med, 2021, 25(14): 7013-7027.

[41] Chan CH, Wu CY, Dubey NK, et al. Modulating redox homeostasis and cellular reprogramming through inhibited methylenetetrahydrofolate dehydrogenase 2 enzymatic activities in lung cancer[J]. Aging (Albany NY), 2020, 12(18): 17930-17947.

[42] Yu C, Yang L, Cai M, et al. Down-regulation of MTHFD2 inhibits NSCLC progression by suppressing cycle-related genes[J]. J Cell Mol Med, 2020, 24(2): 1568-1577.

[43] Bach DH, Kim D, Bae SY, et al. Targeting Nicotinamide N-Methyltransferase and miR-449a in EGFR-TKI-Resistant Non-Small-Cell Lung Cancer Cells[J]. Mol Ther Nucleic Acids, 2018, 11: 455-467.

[44] Sartini D, Morganti S, Guidi E, et al. Nicotinamide N-methyltransferase in non-small cell lung cancer: promising results for targeted anti-cancer therapy[J]. Cell Biochem Biophys, 2013, 67(3): 865-873.

[45] Sartini D, Seta R, Pozzi V, et al. Role of nicotinamide N-methyltransferase in non-small cell lung cancer: in vitro effect of shRNA-mediated gene silencing on tumourigenicity[J]. Biol Chem, 2015, 396(3): 225-234.

[46] Liu Y, Wu L, Li K, et al. Ornithine aminotransferase promoted the proliferation and metastasis of non-small cell lung cancer via upregulation of miR-21[J]. J Cell Physiol, 2019, 234(8): 12828-12838.

[47] Cao Q, Liu Y, Wu Y, et al. Profilin 2 promotes growth, metastasis, and angiogenesis of small cell lung cancer through cancer-derived exosomes[J]. Aging (Albany NY), 2020, 12(24): 25981-25999.

[48] Tang YN, Ding WQ, Guo XJ, et al. Epigenetic regulation of Smad2 and Smad3 by profilin-2 promotes lung cancer growth and metastasis[J]. Nat Commun, 2015, 6: 8230.

[49] Meng X, Sun R, Wang W, et al. ADFP promotes cell proliferation in lung adenocarcinoma via Akt phosphorylation[J]. J Cell Mol Med, 2021, 25(2): 827-839.

[50] Liu C, Yao Y, Wang W. Pentraxin-3 as a prognostic marker in patients with small-cell lung cancer[J]. Med Oncol, 2014, 31(10): 207.

[51] Li Y, Song X, Niu J, et al. Pentraxin 3 acts as a functional effector of Akt/NF-κB signaling to modulate the progression and cisplatin-resistance in non-small cell lung cancer[J]. Arch Biochem Biophys, 2021, 701: 108818.

[52] Hu FQ, Qiao T, Xie X, et al. Knockdown of the inflammatory factor pentraxin-3 suppresses growth and invasion of lung adenocarcinoma through the AKT and NF-kappa B pathways[J]. J Biol Regul Homeost Agents, 2014, 28(4): 649-657.

[53] Chen X, Shao W, Huang H, et al. Overexpression of RCN1 correlates with poor prognosis and progression in non-small cell lung cancer[J]. Hum Pathol, 2019, 83: 140-148.

[54] Fu H, Chen R, Wang Y, et al. Reticulocalbin 1 is required for proliferation and migration of non-small cell lung cancer cells regulated by osteoblast-conditioned medium[J]. J Cell Mol Med, 2021, 25(24): 11198-11211.

[55] Hsu YL, Hung JY, Liang YY, et al. S100P interacts with integrin α7 and increases cancer cell migration and invasion in lung cancer[J]. Oncotarget, 2015, 6(30): 29585-29598.

[56] Kong HJ, Kwon EJ, Kwon OS, et al. Crosstalk between YAP and TGFβ regulates SERPINE1 expression in mesenchymal lung cancer cells[J]. Int J Oncol, 2021, 58(1): 111-121.

[57] Parekh K, Ramachandran S, Cooper J, et al. Tenascin-C, over expressed in lung cancer down regulates effector functions of tumor infiltrating lymphocytes[J]. Lung Cancer, 2005, 47(1): 17-29.

[58] Sun Z, Velázquez-Quesada I, Murdamoothoo D, et al. Tenascin-C increases lung metastasis by impacting blood vessel invasions[J]. Matrix Biol, 2019, 83: 26-47.

[59] Ho CC, Liao WY, Wang CY, et al. TREM-1 expression in tumor-associated macrophages and clinical outcome in lung cancer[J]. Am J Respir Crit Care Med, 2008, 177(7): 763-770.

[60] Sigalov AB. A novel ligand-independent peptide inhibitor of TREM-1 suppresses tumor growth in human lung cancer xenografts and prolongs survival of mice with lipopolysaccharide-induced septic shock[J]. Int Immunopharmacol, 2014, 21(1): 208-219.

[61] Guo T, Kong J, Liu Y, et al. Transcriptional activation of NANOG by YBX1 promotes lung cancer stem-like properties and metastasis[J]. Biochem Biophys Res Commun, 2017, 487(1): 153-159.

[62] Cui Y, Li F, Xie Q, et al. YBX1 mediates autophagy by targeting p110β and decreasing the sensitivity to cisplatin in NSCLC[J]. Cell Death Dis, 2020, 11(6): 476.

[63] Hu B, Zhu X, Lu J. Cathepsin A knockdown decreases the proliferation and invasion of A549 lung adenocarcinoma cells[J]. Mol Med Rep, 2020, 21(6): 2553-2559.

[64] Xu P, Wang L, Xie X, et al. Hsa_circ_0001869 promotes NSCLC progression via sponging miR-638 and enhancing FOSL2 expression[J]. Aging (Albany NY), 2020, 12(23): 23836-23848.

[65] Yin J, Hu W, Fu W, et al. HGF/MET Regulated Epithelial-Mesenchymal Transitions And Metastasis By FOSL2 In Non-Small Cell Lung Cancer[J]. Onco Targets Ther, 2019, 12: 9227-9237.

[66] Liu C, Tang J, Duan X, et al. DDX10 promotes human lung carcinoma proliferation by U3 small nucleolar ribonucleoprotein IMP4[J]. Thorac Cancer, 2021, 12(12): 1873-1880.

[67] Yao S, Huang HY, Han X, et al. Keratin 14-high subpopulation mediates lung cancer metastasis potentially through Gkn1 upregulation[J]. Oncogene, 2019, 38(36): 6354-6369.

[68] Tsubokawa F, Nishisaka T, Takeshima Y, et al. Heterogeneity of expression of cytokeratin subtypes in squamous cell carcinoma of the lung: with special reference to CK14 overexpression in cancer of high-proliferative and lymphogenous metastatic potential[J]. Pathol Int, 2002, 52(4): 286-293.

[69] Zeltz C, Pasko E, Cox TR, et al. LOXL1 Is Regulated by Integrin α11 and Promotes Non-Small Cell Lung Cancer Tumorigenicity[J]. Cancers (Basel), 2019, 11(5).

[70] Zhu J, Wu C, Li H, et al. DACH1 inhibits the proliferation and invasion of lung adenocarcinoma through the downregulation of peroxiredoxin 3[J]. Tumour Biol, 2016, 37(7): 9781-9788.

[71] Zheng J, Guo X, Nakamura Y, et al. Overexpression of PRDX4 Modulates Tumor Microenvironment and Promotes Urethane-Induced Lung Tumorigenesis[J]. Oxid Med Cell Longev, 2020, 2020: 8262730.

[72] Hwang JA, Song JS, Yu DY, et al. Peroxiredoxin 4 as an independent prognostic marker for survival in patients with early-stage lung squamous cell carcinoma[J]. Int J Clin Exp Pathol, 2015, 8(6): 6627-6635.

[73] Mizutani K, Guo X, Shioya A, et al. The impact of PRDX4 and the EGFR mutation status on cellular proliferation in lung adenocarcinoma[J]. Int J Med Sci, 2019, 16(9): 1199-1206.

[74] Zhang Y, Wei Y, Jiang B, et al. Scavenger Receptor A1 Prevents Metastasis of Non-Small Cell Lung Cancer via Suppression of Macrophage Serum Amyloid A1[J]. Cancer Res, 2017, 77(7): 1586-1598.

[75] Zhang J, Song F, Zhao X, et al. EGFR modulates monounsaturated fatty acid synthesis through phosphorylation of SCD1 in lung cancer[J]. Mol Cancer, 2017, 16(1): 127.

[76] Scaglia N, Igal RA. Inhibition of Stearoyl-CoA Desaturase 1 expression in human lung adenocarcinoma cells impairs tumorigenesis[J]. Int J Oncol, 2008, 33(4): 839-850.

[77] Ma YS, Hou LK, Yao SH, et al. Elevated Stratifin promotes cisplatin-based chemotherapy failure and poor prognosis in non-small cell lung cancer[J]. Mol Ther Oncolytics, 2021, 22: 326-335.

[78] Shiba-Ishii A, Kim Y, Shiozawa T, et al. Stratifin accelerates progression of lung adenocarcinoma at an early stage[J]. Mol Cancer, 2015, 14: 142.

[79] Kim Y, Shiba-Ishii A, Nakagawa T, et al. Stratifin regulates stabilization of receptor tyrosine kinases via interaction with ubiquitin-specific protease 8 in lung adenocarcinoma[J]. Oncogene, 2018, 37(40): 5387-5402.

[80] Chen PM, Wu TC, Wang YC, et al. Activation of NF-κB by SOD2 promotes the aggressiveness of lung adenocarcinoma by modulating NKX2-1-mediated IKKβ expression[J]. Carcinogenesis, 2013, 34(11): 2655-2663.

[81] Chen Y, Yang S, Zhou H, et al. PRDX2 Promotes the Proliferation and Metastasis of Non-Small Cell Lung Cancer In Vitro and In Vivo[J]. Biomed Res Int, 2020, 2020: 8359860.

[82] Jing X, Du L, Niu A, et al. Silencing of PRDX2 Inhibits the Proliferation and Invasion of Non-Small Cell Lung Cancer Cells[J]. Biomed Res Int, 2020, 2020: 1276328.

[83] Rousalova I, Krepela E, Prochazka J, et al. Expression of proteinase inhibitor-9/serpinB9 in non-small cell lung carcinoma cells and tissues[J]. Int J Oncol, 2010, 36(1): 275-283.

[84] Peng J, Liu HZ, Zhong J, et al. MicroRNA‑187 is an independent prognostic factor in lung cancer and promotes lung cancer cell invasion via targeting of PTRF[J]. Oncol Rep, 2016, 36(5): 2609-2618.

[85] Cai Y, Ruan J, Yao X, et al. MicroRNA-187 modulates epithelial-mesenchymal transition by targeting PTRF in non-small cell lung cancer[J]. Oncol Rep, 2017, 37(5): 2787-2794.

[86] Han Z, Wang T, Han S, et al. Low-expression of TMEM100 is associated with poor prognosis in non-small-cell lung cancer[J]. Am J Transl Res, 2017, 9(5): 2567-2578.

[87] Wang Y, Ha M, Li M, et al. Histone deacetylase 6-mediated downregulation of TMEM100 expedites the development and progression of non-small cell lung cancer[J]. Hum Cell, 2021.

[88] Ma J, Yan T, Bai Y, et al. TMEM100 negatively regulated by microRNA‑106b facilitates cellular apoptosis by suppressing survivin expression in NSCLC[J]. Oncol Rep, 2021, 46(2).

[89] Zeng GQ, Yi H, Zhang PF, et al. The function and significance of SELENBP1 downregulation in human bronchial epithelial carcinogenic process[J]. PLoS One, 2013, 8(8): e71865.

[90] Caswell DR, Chuang CH, Ma RK, et al. Tumor Suppressor Activity of Selenbp1, a Direct Nkx2-1 Target, in Lung Adenocarcinoma[J]. Mol Cancer Res, 2018, 16(11): 1737-1749.

[91] Lou Z, Lin W, Zhao H, et al. Alkaline phosphatase downregulation promotes lung adenocarcinoma metastasis via the c-Myc/RhoA axis[J]. Cancer Cell Int, 2021, 21(1): 217.

[92] Wang M, Zhang G, Zhang Y, et al. Fibrinogen Alpha Chain Knockout Promotes Tumor Growth and Metastasis through Integrin-AKT Signaling Pathway in Lung Cancer[J]. Mol Cancer Res, 2020, 18(7): 943-954.

[93] Jacquet M, Hervouet E, Baudu T, et al. GABARAPL1 Inhibits EMT Signaling through SMAD-Tageted Negative Feedback[J]. Biology (Basel), 2021, 10(10).

[94] Cheng H, Wang A, Meng J, et al. Enhanced metastasis in RNF13 knockout mice is mediated by a reduction in GM-CSF levels[J]. Protein Cell, 2015, 6(10): 746-756.

[95] Muthu M, Kumar R, Syed Khaja AS, et al. GLUL Ablation Can Confer Drug Resistance to Cancer Cells via a Malate-Aspartate Shuttle-Mediated Mechanism[J]. Cancers (Basel), 2019, 11(12).

[96] Wang L, Peng W, Wu T, et al. Increased glutamine anabolism sensitizes non-small cell lung cancer to gefitinib treatment[J]. Cell Death Discov, 2018, 4: 24.

[97] Qi LN, Ma L, Wu FX, et al. S100P as a novel biomarker of microvascular invasion and portal vein tumor thrombus in hepatocellular carcinoma[J]. Hepatol Int, 2021, 15(1): 114-126.

[98] Yuan RH, Chang KT, Chen YL, et al. S100P expression is a novel prognostic factor in hepatocellular carcinoma and predicts survival in patients with high tumor stage or early recurrent tumors[J]. PLoS One, 2013, 8(6): e65501.

[99] Hwang HS, An J, Kang HJ, et al. Prognostic Molecular Indices of Resectable Hepatocellular Carcinoma: Implications of S100P for Early Recurrence[J]. Ann Surg Oncol, 2021, 28(11): 6466-6478.

[100] Lv X, Yu H, Zhang Q, et al. SRXN1 stimulates hepatocellular carcinoma tumorigenesis and metastasis through modulating ROS/p65/BTG2 signalling[J]. J Cell Mol Med, 2020, 24(18): 10714-10729.

[101] Rao QW, Zhang SL, Guo MZ, et al. Sulfiredoxin-1 is a promising novel prognostic biomarker for hepatocellular carcinoma[J]. Cancer Med, 2020, 9(22): 8318-8332.

[102] Dong Y, Sun X, Zhang K, et al. Type IIA topoisomerase (TOP2A) triggers epithelial-mesenchymal transition and facilitates HCC progression by regulating Snail expression[J]. Bioengineered, 2021, 12(2): 12967-12979.

[103] Wong N, Yeo W, Wong WL, et al. TOP2A overexpression in hepatocellular carcinoma correlates with early age onset, shorter patients survival and chemoresistance[J]. Int J Cancer, 2009, 124(3): 644-652.

[104] Zhou SF, Mo FR, Bin YH, et al. Serum immunoreactivity of SMP30 and its tissues expression in hepatocellular carcinoma[J]. Clin Biochem, 2011, 44(4): 331-336.

[105] Mo Z, Zheng S, Lv Z, et al. Senescence marker protein 30 (SMP30) serves as a potential prognostic indicator in hepatocellular carcinoma[J]. Sci Rep, 2016, 6: 39376.

[106] Zhang SC, Liang MK, Huang GL, et al. Inhibition of SMP30 gene expression influences the biological characteristics of human Hep G2 cells[J]. Asian Pac J Cancer Prev, 2014, 15(3): 1193-1196.

[107] Li A, Yan Q, Zhao X, et al. Decreased expression of PBLD correlates with poor prognosis and functions as a tumor suppressor in human hepatocellular carcinoma[J]. Oncotarget, 2016, 7(1): 524-537.

[108] Wu J, Niu Q, Yuan J, et al. Novel compound cedrelone inhibits hepatocellular carcinoma progression via PBLD and Ras/Rap1[J]. Exp Ther Med, 2019, 18(6): 4209-4220.

[109] Wang J, Li T, Ma L, et al. NDRG2 inhibition facilitates angiogenesis of hepatocellular carcinoma[J]. Open Med (Wars), 2021, 16(1): 742-748.

[110] Lee DC, Kang YK, Kim WH, et al. Functional and clinical evidence for NDRG2 as a candidate suppressor of liver cancer metastasis[J]. Cancer Res, 2008, 68(11): 4210-4220.

[111] Zheng J, Li Y, Yang J, et al. NDRG2 inhibits hepatocellular carcinoma adhesion, migration and invasion by regulating CD24 expression[J]. BMC Cancer, 2011, 11: 251:251-259.

[112] Chen GL, Ye T, Chen HL, et al. Xanthine dehydrogenase downregulation promotes TGFβ signaling and cancer stem cell-related gene expression in hepatocellular carcinoma[J]. Oncogenesis, 2017, 6(9): e382.

[113] Qin YJ, Lin TY, Lin XL, et al. Loss of PDK4 expression promotes proliferation, tumorigenicity, motility and invasion of hepatocellular carcinoma cells[J]. J Cancer, 2020, 11(15): 4397-4405.

[114] Yang C, Wang S, Ruan H, et al. Downregulation of PDK4 Increases Lipogenesis and Associates with Poor Prognosis in Hepatocellular Carcinoma[J]. J Cancer, 2019, 10(4): 918-926.

[115] Si T, Ning X, Zhao H, et al. microRNA-9-5p regulates the mitochondrial function of hepatocellular carcinoma cells through suppressing PDK4[J]. Cancer Gene Ther, 2021, 28(6): 706-718.

[116] Tu K, Liu Z, Yao B, et al. BCL-3 promotes the tumor growth of hepatocellular carcinoma by regulating cell proliferation and the cell cycle through cyclin D1[J]. Oncol Rep, 2016, 35(4): 2382-2390.

[117] Huang Y, Yang X, Meng Y, et al. The hepatic senescence-associated secretory phenotype promotes hepatocarcinogenesis through Bcl3-dependent activation of macrophages[J]. Cell Biosci, 2021, 11(1): 173.

[118] Ke AW, Shi GM, Zhou J, et al. CD151 amplifies signaling by integrin α6β1 to PI3K and induces the epithelial-mesenchymal transition in HCC cells[J]. Gastroenterology, 2011, 140(5): 1629-1641.e1615.

[119] Shi GM, Ke AW, Zhou J, et al. CD151 modulates expression of matrix metalloproteinase 9 and promotes neoangiogenesis and progression of hepatocellular carcinoma[J]. Hepatology, 2010, 52(1): 183-196.

[120] Wadkin JCR, Patten DA, Kamarajah SK, et al. CD151 supports VCAM-1-mediated lymphocyte adhesion to liver endothelium and is upregulated in chronic liver disease and hepatocellular carcinoma[J]. Am J Physiol Gastrointest Liver Physiol, 2017, 313(2): G138-g149.

[121] Ke AW, Shi GM, Zhou J, et al. Role of overexpression of CD151 and/or c-Met in predicting prognosis of hepatocellular carcinoma[J]. Hepatology, 2009, 49(2): 491-503.

[122] Lin Q, Peng S, Yang Y. Inhibition of CD9 expression reduces the metastatic capacity of human hepatocellular carcinoma cell line MHCC97-H[J]. Int J Oncol, 2018, 53(1): 266-274.

[123] Pan JJ, Ge YS, Xu GL, et al. The expression of chitinase 3-like 1: a novel prognostic predictor for hepatocellular carcinoma[J]. J Cancer Res Clin Oncol, 2013, 139(6): 1043-1054.

[124] Qiu QC, Wang L, Jin SS, et al. CHI3L1 promotes tumor progression by activating TGF-β signaling pathway in hepatocellular carcinoma[J]. Sci Rep, 2018, 8(1): 15029.

[125] Zhang C, Cui X, Feng L, et al. The deficiency of FKBP-5 inhibited hepatocellular progression by increasing the infiltration of distinct immune cells and inhibiting obesity-associated gut microbial metabolite[J]. J Gastrointest Oncol, 2021, 12(2): 711-721.

[126] Wang CH, Zhu XD, Ma DN, et al. Flot2 promotes tumor growth and metastasis through modulating cell cycle and inducing epithelial-mesenchymal transition of hepatocellular carcinoma[J]. Am J Cancer Res, 2017, 7(5): 1068-1083.

[127] Ye QH, Zhu WW, Zhang JB, et al. GOLM1 Modulates EGFR/RTK Cell-Surface Recycling to Drive Hepatocellular Carcinoma Metastasis[J]. Cancer Cell, 2016, 30(3): 444-458.

[128] Chen MH, Jan YH, Chang PM, et al. Expression of GOLM1 correlates with prognosis in human hepatocellular carcinoma[J]. Ann Surg Oncol, 2013, 20 Suppl 3: S616-624.

[129] Chen J, Lin Z, Liu L, et al. GOLM1 exacerbates CD8(+) T cell suppression in hepatocellular carcinoma by promoting exosomal PD-L1 transport into tumor-associated macrophages[J]. Signal Transduct Target Ther, 2021, 6(1): 397.

[130] Ke MY, Xu T, Fang Y, et al. Liver fibrosis promotes immune escape in hepatocellular carcinoma via GOLM1-mediated PD-L1 upregulation[J]. Cancer Lett, 2021, 513: 14-25.

[131] Suzuki S, Pitchakarn P, Ogawa K, et al. Expression of glutathione peroxidase 2 is associated with not only early hepatocarcinogenesis but also late stage metastasis[J]. Toxicology, 2013, 311(3): 115-123.

[132] Gwak GY, Yoon JH, Kim KM, et al. Hypoxia stimulates proliferation of human hepatoma cells through the induction of hexokinase II expression[J]. J Hepatol, 2005, 42(3): 358-364.

[133] Meng YM, Jiang X, Zhao X, et al. Hexokinase 2-driven glycolysis in pericytes activates their contractility leading to tumor blood vessel abnormalities[J]. Nat Commun, 2021, 12(1): 6011.

[134] Fu SJ, Shen SL, Li SQ, et al. Hornerin promotes tumor progression and is associated with poor prognosis in hepatocellular carcinoma[J]. BMC Cancer, 2018, 18(1): 815.

[135] He Y, Zheng Z, Xu Y, et al. Over-expression of IMPDH2 is associated with tumor progression and poor prognosis in hepatocellular carcinoma[J]. Am J Cancer Res, 2018, 8(8): 1604-1614.

[136] Matsumoto K, Noda T, Kobayashi S, et al. Inhibition of glycolytic activator PFKFB3 suppresses tumor growth and induces tumor vessel normalization in hepatocellular carcinoma[J]. Cancer Lett, 2021, 500: 29-40.

[137] Shi WK, Zhu XD, Wang CH, et al. PFKFB3 blockade inhibits hepatocellular carcinoma growth by impairing DNA repair through AKT[J]. Cell Death Dis, 2018, 9(4): 428.

[138] Chen G, Li X, Yang J, et al. Prognostic significance of cyclooxygenase-2 expression in patients with hepatocellular carcinoma: a meta-analysis[J]. Arch Med Sci, 2016, 12(5): 1110-1117.

[139] Chen H, Cai W, Chu ESH, et al. Hepatic cyclooxygenase-2 overexpression induced spontaneous hepatocellular carcinoma formation in mice[J]. Oncogene, 2017, 36(31): 4415-4426.

[140] Ogunwobi OO, Wang T, Zhang L, et al. Cyclooxygenase-2 and Akt mediate multiple growth-factor-induced epithelial-mesenchymal transition in human hepatocellular carcinoma[J]. J Gastroenterol Hepatol, 2012, 27(3): 566-578.

[141] Dong W, Dai ZH, Liu FC, et al. The RNA-binding protein RBM3 promotes cell proliferation in hepatocellular carcinoma by regulating circular RNA SCD-circRNA 2 production[J]. EBioMedicine, 2019, 45: 155-167.

[142] Ko E, Kim JS, Bae JW, et al. SERPINA3 is a key modulator of HNRNP-K transcriptional activity against oxidative stress in HCC[J]. Redox Biol, 2019, 24: 101217.

[143] Jin Y, Liang ZY, Zhou WX, et al. Expression, clinicopathologic and prognostic significance of plasminogen activator inhibitor 1 in hepatocellular carcinoma[J]. Cancer Biomark, 2020, 27(3): 285-293.

[144] Geis T, Döring C, Popp R, et al. HIF-2alpha-dependent PAI-1 induction contributes to angiogenesis in hepatocellular carcinoma[J]. Exp Cell Res, 2015, 331(1): 46-57.

[145] Huang K, Xie W, Wang S, et al. High SPINK1 Expression Predicts Poor Prognosis and Promotes Cell Proliferation and Metastasis of Hepatocellular Carcinoma[J]. J Invest Surg, 2021, 34(9): 1011-1020.

[146] Ying HY, Gong CJ, Feng Y, et al. Serine protease inhibitor Kazal type 1 (SPINK1) downregulates E-cadherin and induces EMT of hepatoma cells to promote hepatocellular carcinoma metastasis via the MEK/ERK signaling pathway[J]. J Dig Dis, 2017, 18(6): 349-358.

[147] Akiel MA, Santhekadur PK, Mendoza RG, et al. Tetraspanin 8 mediates AEG-1-induced invasion and metastasis in hepatocellular carcinoma cells[J]. FEBS Lett, 2016, 590(16): 2700-2708.

[148] Kanetaka K, Sakamoto M, Yamamoto Y, et al. Possible involvement of tetraspanin CO-029 in hematogenous intrahepatic metastasis of liver cancer cells[J]. J Gastroenterol Hepatol, 2003, 18(11): 1309-1314.

[149] Kanetaka K, Sakamoto M, Yamamoto Y, et al. Overexpression of tetraspanin CO-029 in hepatocellular carcinoma[J]. J Hepatol, 2001, 35(5): 637-642.

[150] Wu MN, Zheng WJ, Ye WX, et al. Oncogenic tuftelin 1 as a potential molecular-targeted for inhibiting hepatocellular carcinoma growth[J]. World J Gastroenterol, 2021, 27(23): 3327-3341.

[151] Dou C, Zhou Z, Xu Q, et al. Hypoxia-induced TUFT1 promotes the growth and metastasis of hepatocellular carcinoma by activating the Ca(2+)/PI3K/AKT pathway[J]. Oncogene, 2019, 38(8): 1239-1255.

[152] Chen YY, Wang WH, Che L, et al. BNIP3L-Dependent Mitophagy Promotes HBx-Induced Cancer Stemness of Hepatocellular Carcinoma Cells via Glycolysis Metabolism Reprogramming[J]. Cancers (Basel), 2020, 12(3).

[153] Yang H, Liu Y, Zhao MM, et al. Therapeutic potential of targeting membrane-spanning proteoglycan SDC4 in hepatocellular carcinoma[J]. Cell Death Dis, 2021, 12(5): 492.

[154] Sun Y, Shi P, Wu Q, et al. MiR-222-3p induced by hepatitis B virus promotes the proliferation and inhibits apoptosis in hepatocellular carcinoma by upregulating THBS1[J]. Hum Cell, 2021, 34(6): 1788-1799.

[155] Fu PY, Hu B, Ma XL, et al. Far upstream element-binding protein 1 facilitates hepatocellular carcinoma invasion and metastasis[J]. Carcinogenesis, 2020, 41(7): 950-960.

[156] Wang J, Zhou Y, Li D, et al. TSPAN31 is a critical regulator on transduction of survival and apoptotic signals in hepatocellular carcinoma cells[J]. FEBS Lett, 2017, 591(18): 2905-2918.

[157] Hsu SH, Wang LT, Chai CY, et al. Aryl hydrocarbon receptor promotes hepatocellular carcinoma tumorigenesis by targeting intestine-specific homeobox expression[J]. Mol Carcinog, 2017, 56(10): 2167-2177.

[158] Zhong W, Liu H, Deng L, et al. HBO1 overexpression is important for hepatocellular carcinoma cell growth[J]. Cell Death Dis, 2021, 12(6): 549.

[159] Gai X, Tu K, Lu Z, et al. MRC2 expression correlates with TGFβ1 and survival in hepatocellular carcinoma[J]. Int J Mol Sci, 2014, 15(9): 15011-15025.

[160] Emma MR, Iovanna JL, Bachvarov D, et al. NUPR1, a new target in liver cancer: implication in controlling cell growth, migration, invasion and sorafenib resistance[J]. Cell Death Dis, 2016, 7(6): e2269.

[161] Li C, Chen J, Li Y, et al. 6-Phosphogluconolactonase Promotes Hepatocellular Carcinogenesis by Activating Pentose Phosphate Pathway[J]. Front Cell Dev Biol, 2021, 9: 753196.

[162] Chen K, Li Z, Zhang M, et al. miR-876 Inhibits EMT and Liver Fibrosis via POSTN to Suppress Metastasis in Hepatocellular Carcinoma[J]. Biomed Res Int, 2020, 2020: 1964219.

[163] Wen Q, Shang J, Mise SRL, et al. [Effects of periostin on hepatocellular carcinoma cells invasion, metastasis and prognosis][J]. Zhonghua Gan Zang Bing Za Zhi, 2019, 27(10): 766-771.

[164] Tang M, Etokidem E, Lai K. The Leloir Pathway of Galactose Metabolism - A Novel Therapeutic Target for Hepatocellular Carcinoma[J]. Anticancer Res, 2016, 36(12): 6265-6271.

[165] Wei X, Li J, Xie H, et al. Proteomics-based identification of the tumor suppressor role of aminoacylase 1 in hepatocellular carcinoma[J]. Cancer Lett, 2014, 351(1): 117-125.

[166] Zhang H, Zhuo C, Zhou D, et al. Association between the expression of carbonic anhydrase II and clinicopathological features of hepatocellular carcinoma[J]. Oncol Lett, 2019, 17(6): 5721-5728.

[167] Zhang C, Wang H, Chen Z, et al. Carbonic anhydrase 2 inhibits epithelial-mesenchymal transition and metastasis in hepatocellular carcinoma[J]. Carcinogenesis, 2018, 39(4): 562-570.

[168] Oya H, Kanda M, Sugimoto H, et al. Dihydropyrimidinase-like 3 is a putative hepatocellular carcinoma tumor suppressor[J]. J Gastroenterol, 2015, 50(5): 590-600.

[169] Liu X, Sui X, Zhang C, et al. Glutathione S-transferase A1 suppresses tumor progression and indicates better prognosis of human primary hepatocellular carcinoma[J]. J Cancer, 2020, 11(1): 83-91.

[170] Jin GZ, Zhang Y, Cong WM, et al. Phosphoglucomutase 1 inhibits hepatocellular carcinoma progression by regulating glucose trafficking[J]. PLoS Biol, 2018, 16(10): e2006483.

[171] Yu M, Pan L, Sang C, et al. Apolipoprotein M could inhibit growth and metastasis of SMMC7721 cells via vitamin D receptor signaling[J]. Cancer Manag Res, 2019, 11: 3691-3701.

[172] Li Z, Lou Y, Tian G, et al. Discovering master regulators in hepatocellular carcinoma: one novel MR, SEC14L2 inhibits cancer cells[J]. Aging (Albany NY), 2019, 11(24): 12375-12411.

[173] Wu YM, Liu CH, Hu RH, et al. Mucin glycosylating enzyme GALNT2 regulates the malignant character of hepatocellular carcinoma by modifying the EGF receptor[J]. Cancer Res, 2011, 71(23): 7270-7279.

[174] Yuan F, Zhang Y, Ma L, et al. Enhanced NOLC1 promotes cell senescence and represses hepatocellular carcinoma cell proliferation by disturbing the organization of nucleolus[J]. Aging Cell, 2017, 16(4): 726-737.

[175] Zhou C, Liu C, Liu W, et al. SLFN11 inhibits hepatocellular carcinoma tumorigenesis and metastasis by targeting RPS4X via mTOR pathway[J]. Theranostics, 2020, 10(10): 4627-4643.

[176] Chen PD, Wu YM, Huang MC. B4GALT1 gene knockdown promotes hepatocellular carcinoma malignancy[J]. Liver Cancer, 2018, 7: 128.

[177] Luo L, Chen L, Ke K, et al. High expression levels of CLEC4M indicate poor prognosis in patients with hepatocellular carcinoma[J]. Oncol Lett, 2020, 19(3): 1711-1720.

[178] Yu Q, Gao K. CLEC4M overexpression inhibits progression and is associated with a favorable prognosis in hepatocellular carcinoma[J]. Mol Med Rep, 2020, 22(3): 2245-2252.

[179] Holstein S, Venz S, Junker H, et al. Nicotinamide N-Methyltransferase and Its Precursor Substrate Methionine Directly and Indirectly Control Malignant Metabolism During Progression of Renal Cell Carcinoma[J]. Anticancer Res, 2019, 39(10): 5427-5436.

[180] Zhang J, Xie XY, Yang SW, et al. Nicotinamide N-methyltransferase protein expression in renal cell cancer[J]. J Zhejiang Univ Sci B, 2010, 11(2): 136-143.

[181] Tang SW, Yang TC, Lin WC, et al. Nicotinamide N-methyltransferase induces cellular invasion through activating matrix metalloproteinase-2 expression in clear cell renal cell carcinoma cells[J]. Carcinogenesis, 2011, 32(2): 138-145.

[182] Erdem M, Erdem S, Sanli O, et al. Up-regulation of TGM2 with ITGB1 and SDC4 is important in the development and metastasis of renal cell carcinoma[J]. Urol Oncol, 2014, 32(1): 25.e13-20.

[183] Zhong Y, Onuki J, Yamasaki T, et al. Genome-wide analysis identifies a tumor suppressor role for aminoacylase 1 in iron-induced rat renal cell carcinoma[J]. Carcinogenesis, 2009, 30(1): 158-164.

[184] Xiao W, Wang X, Wang T, et al. HAO2 inhibits malignancy of clear cell renal cell carcinoma by promoting lipid catabolic process[J]. J Cell Physiol, 2019, 234(12): 23005-23016.

[185] Xiong Z, Yuan C, Shi J, et al. Restoring the epigenetically silenced PCK2 suppresses renal cell carcinoma progression and increases sensitivity to sunitinib by promoting endoplasmic reticulum stress[J]. Theranostics, 2020, 10(25): 11444-11461.

[186] Li B, Qiu B, Lee DS, et al. Fructose-1,6-bisphosphatase opposes renal carcinoma progression[J]. Nature, 2014, 513(7517): 251-255.

[187] Weber A, Kristiansen I, Johannsen M, et al. The FUSE binding proteins FBP1 and FBP3 are potential c-myc regulators in renal, but not in prostate and bladder cancer[J]. BMC Cancer, 2008, 8: 369.

[188] Wen YC, Lin YW, Chu CY, et al. Melatonin-triggered post-transcriptional and post-translational modifications of ADAMTS1 coordinately retard tumorigenesis and metastasis of renal cell carcinoma[J]. J Pineal Res, 2020, 69(2): e12668.

[189] Wang J, Lei W, Li G, et al. CD151 promotes proliferation and migration of SK-NEP-1 cells via the GSK-3β/P21/cyclinD signaling pathway[J]. Pathol Res Pract, 2019, 215(2): 329-334.

[190] Yoo SH, Lee K, Chae JY, et al. CD151 expression can predict cancer progression in clear cell renal cell carcinoma[J]. Histopathology, 2011, 58(2): 191-197.

[191] Hoefflin R, Harlander S, Schäfer S, et al. HIF-1α and HIF-2α differently regulate tumour development and inflammation of clear cell renal cell carcinoma in mice[J]. Nat Commun, 2020, 11(1): 4111.

[192] Klatte T, Seligson DB, Riggs SB, et al. Hypoxia-inducible factor 1 alpha in clear cell renal cell carcinoma[J]. Clin Cancer Res, 2007, 13(24): 7388-7393.

[193] Kondo Y, Hamada J, Kobayashi C, et al. Over expression of hypoxia-inducible factor-1alpha in renal and bladder cancer cells increases tumorigenic potency[J]. J Urol, 2005, 173(5): 1762-1766.

[194] Kanno T, Kamba T, Yamasaki T, et al. JunB promotes cell invasion and angiogenesis in VHL-defective renal cell carcinoma[J]. Oncogene, 2012, 31(25): 3098-3110.

[195] Li S, Cheng Y, Cheng G, et al. High SAA1 Expression Predicts Advanced Tumors in Renal Cancer[J]. Front Oncol, 2021, 11: 649761.

[196] Liu H, Chen A, Guo F, et al. Influence of osteopontin short hairpin RNA on the proliferation and invasion of human renal cancer cells[J]. J Huazhong Univ Sci Technolog Med Sci, 2010, 30(1): 61-68.

[197] Rabjerg M, Bjerregaard H, Halekoh U, et al. Molecular characterization of clear cell renal cell carcinoma identifies CSNK2A1, SPP1 and DEFB1 as promising novel prognostic markers[J]. Apmis, 2016, 124(5): 372-383.

[198] Matusan K, Dordevic G, Stipic D, et al. Osteopontin expression correlates with prognostic variables and survival in clear cell renal cell carcinoma[J]. J Surg Oncol, 2006, 94(4): 325-331.

[199] Wan YP, Xi M, He HC, et al. Expression and Clinical Significance of SOX9 in Renal Cell Carcinoma, Bladder Cancer and Penile Cancer[J]. Oncol Res Treat, 2017, 40(1-2): 15-20.

[200] Li XL, Chen XQ, Zhang MN, et al. SOX9 was involved in TKIs resistance in renal cell carcinoma via Raf/MEK/ERK signaling pathway[J]. Int J Clin Exp Pathol, 2015, 8(4): 3871-3881.

[201] Hedrick E, Lee SO, Kim G, et al. Nuclear Receptor 4A1 (NR4A1) as a Drug Target for Renal Cell Adenocarcinoma[J]. PLoS One, 2015, 10(6): e0128308.

[202] Abu Aboud O, Chen CH, Senapedis W, et al. Dual and Specific Inhibition of NAMPT and PAK4 By KPT-9274 Decreases Kidney Cancer Growth[J]. Mol Cancer Ther, 2016, 15(9): 2119-2129.

[203] Danilin S, Amiable C, Kaminski PA, et al. Oncogenic versus tumour suppressor potential of the new N-hydrolase DNPH1 in prostate and kidney cancers[J]. European Urology, Supplements, 2015, 14(2): e286.

[204] Dong X, Kong C, Zhang Z, et al. hZIP1 that is down-regulated in clear cell renal cell carcinoma is negatively associated with the malignant potential of the tumor[J]. Urol Oncol, 2014, 32(6): 885-892.

[205] Ye H, Wang WG, Cao J, et al. SPARCL1 suppresses cell migration and invasion in renal cell carcinoma[J]. Mol Med Rep, 2017, 16(5): 7784-7790.

[206] Chen J, Gu Y, Shen W. MicroRNA-21 functions as an oncogene and promotes cell proliferation and invasion via TIMP3 in renal cancer[J]. Eur Rev Med Pharmacol Sci, 2017, 21(20): 4566-4576.

[207] Mao S, Zhang D, Chen L, et al. FKBP51 promotes invasion and migration by increasing the autophagic degradation of TIMP3 in clear cell renal cell carcinoma[J]. Cell Death Dis, 2021, 12(10): 899.

[208] Li W, Song YY, Rao T, et al. CircCSNK1G3 up-regulates miR-181b to promote growth and metastasis via TIMP3-mediated epithelial to mesenchymal transitions in renal cell carcinoma[J]. J Cell Mol Med, 2021.

[209] Ochocki JD, Khare S, Hess M, et al. Arginase 2 Suppresses Renal Carcinoma Progression via Biosynthetic Cofactor Pyridoxal Phosphate Depletion and Increased Polyamine Toxicity[J]. Cell Metab, 2018, 27(6): 1263-1280.e1266.

[210] Kummer NT, Nowicki TS, Azzi JP, et al. Arachidonate 5 lipoxygenase expression in papillary thyroid carcinoma promotes invasion via MMP-9 induction[J]. J Cell Biochem, 2012, 113(6): 1998-2008.

[211] Collina F, La Sala L, Liotti F, et al. AXL Is a Novel Predictive Factor and Therapeutic Target for Radioactive Iodine Refractory Thyroid Cancer[J]. Cancers (Basel), 2019, 11(6).

[212] Avilla E, Guarino V, Visciano C, et al. Activation of TYRO3/AXL tyrosine kinase receptors in thyroid cancer[J]. Cancer Res, 2011, 71(5): 1792-1804.

[213] Ito T, Ito M, Naito S, et al. Expression of the Axl receptor tyrosine kinase in human thyroid carcinoma[J]. Thyroid, 1999, 9(6): 563-567.

[214] Zhu X, Bai Q, Lu Y, et al. Expression and function of CXCL12/CXCR4/CXCR7 in thyroid cancer[J]. Int J Oncol, 2016, 48(6): 2321-2329.

[215] Zheng X, Rui S, Wang XF, et al. circPVT1 regulates medullary thyroid cancer growth and metastasis by targeting miR-455-5p to activate CXCL12/CXCR4 signaling[J]. J Exp Clin Cancer Res, 2021, 40(1): 157.

[216] Lin Y, Ma Q, Li L, et al. The CXCL12-CXCR4 axis promotes migration, invasiveness, and EMT in human papillary thyroid carcinoma B-CPAP cells via NF-κB signaling[J]. Biochem Cell Biol, 2018, 96(5): 619-626.

[217] Gao Z, Yu F, Jia H, et al. FK506-binding protein 5 promotes the progression of papillary thyroid carcinoma[J]. J Int Med Res, 2021, 49(4): 3000605211008325.

[218] He J, Zhou M, Li X, et al. SLC34A2 simultaneously promotes papillary thyroid carcinoma growth and invasion through distinct mechanisms[J]. Oncogene, 2020, 39(13): 2658-2675.

[219] Hayase S, Sasaki Y, Matsubara T, et al. Expression of stanniocalcin 1 in thyroid side population cells and thyroid cancer cells[J]. Thyroid, 2015, 25(4): 425-436.

[220] Dai D, Wang Q, Li X, et al. Klotho inhibits human follicular thyroid cancer cell growth and promotes apoptosis through regulation of the expression of stanniocalcin-1[J]. Oncol Rep, 2016, 35(1): 552-558.

[221] Ma X, Wei J, Zhang L, et al. miR-486-5p inhibits cell growth of papillary thyroid carcinoma by targeting fibrillin-1[J]. Biomed Pharmacother, 2016, 80: 220-226.

[222] Yasuoka H, Kodama R, Hirokawa M, et al. Neuropilin-2 expression in papillary thyroid carcinoma: correlation with VEGF-D expression, lymph node metastasis, and VEGF-D-induced aggressive cancer cell phenotype[J]. J Clin Endocrinol Metab, 2011, 96(11): E1857-1861.

[223] Tu DG, Chang WW, Jan MS, et al. Promotion of metastasis of thyroid cancer cells via NRP-2-mediated induction[J]. Oncol Lett, 2016, 12(5): 4224-4230.

[224] Lee G, Kang YE, Oh C, et al. Neuropilin-2 promotes growth and progression of papillary thyroid cancer cells[J]. Auris Nasus Larynx, 2020, 47(5): 870-880.

[225] Huang Z, Xiao F, Zhang F, et al. ApoA1 Promotes Thyroid Carcinoma Metastasis by Modulating EMT via Wnt/beta-catenin Signaling Pathway[J]. Acta Medicinae Universitatis Scientiae et Technologiae Huazhong, 2019, 48(1): 9-14.

[226] Onda M, Akaishi J, Asaka S, et al. Decreased expression of haemoglobin beta (HBB) gene in anaplastic thyroid cancer and recovery of its expression inhibits cell growth[J]. Br J Cancer, 2005, 92(12): 2216-2224.
